# Avian influenza overview December 2025–February 2026

**DOI:** 10.2903/j.efsa.2026.10015

**Published:** 2026-03-27

**Authors:** European Food Safety Authority (EFSA), European Centre for Disease Prevention and Control (ECDC), European Union Reference Laboratory for Avian Influenza (EURL), Cyril Barbezange, Hubert Buczkowski, Mariette Ducatez, Alice Fusaro, Jose L. Gonzales, Thijs Kuiken, Gražina Mirinavičiūtė, Karl Ståhl, Christoph Staubach, Olov Svartström, Calogero Terregino, Katriina Willgert, Evelyn Alarcón, Lisa Kohnle

**Keywords:** avian influenza, captive birds, HPAI, humans, monitoring, poultry, wild birds

## Abstract

Between 29 November 2025 and 27 February 2026, 2514 highly pathogenic avian influenza (HPAI) A(H5) virus detections were reported in domestic (406) and wild (2108) birds in 32 countries in Europe. Albeit still at high levels after the peak was reached at the beginning of the current reporting period, the weekly number of detections has since been declining. Waterfowl species were affected to a greater extent than in previous years, and more than 90% of detections in poultry were due to primary introduction from wild birds. As in previous years, a large number of backyard farms and several zoos were affected. High viral circulation in wild birds resulted in a slight increase in detections in mammals in Europe, with the first potential spillover event from wild birds to dairy cattle suggested in the Netherlands. Between 29 November 2025 and 27 February 2026, 10 cases of avian influenza virus infection were reported in humans (none of which were fatal) in two countries: Cambodia (one A(H5N1) case) and China (eight A(H9N2) cases and one A(H10N3) case). Most human cases reported exposure to poultry or a poultry environment prior to detection or onset of illness. The current high level of avian influenza virus circulation in bird populations increases the risk of human exposure to infected animals, although human infections remain rare and no instance of human‐to‐human transmission has been documented so far. The risk posed by avian A(H5N1) clade 2.3.4.4b influenza viruses currently circulating in Europe remains low for the general public in the European Union/European Economic Area and low‐to‐moderate for those occupationally or otherwise exposed to infected animals or contaminated environments.

**Requestor:** European Commission

**Question number:** EFSA-Q-2026-00135 and Commission request 280 to ECDC (SANTE.B.2/IK/mo (2023)2182203)

**Acknowledgements:** In addition to the listed authors, EFSA, ECDC and the EURL wish to thank the Member State representatives who provided epidemiological data on avian influenza outbreaks or shared sequence data: Lika Aldin and Erga Shkodra (Albania); Andrea Höflechner-Pöltl, Beate Liehl, Sandra Revilla-Fernández and Irene Zimpernik (Austria); Mieke Steensels and Steven Van Borm (Belgium); Emiliya Ivanova (Bulgaria); Vladimir Savic (Croatia); Vasiliki Christodoulou (Cyprus); Klára Jelínková and Alexander Nagy (Czechia); Louise Kathrine Due Fensholdt, Lone Thing Mortensen, Pernille Dahl Nielsen, Charlotte Kristiane Hjulsager, Yuan Liang and Heidi Huus Petersen (Denmark); Kärt Jaarma, Imbi Nurmoja (Estonia); Tuija Gadd, Ari Kauppinen and Niina Tammiranta (Finland); François-Xavier Briand, Béatrice Grasland, Éric Niquex, Audrey Schmitz and Angela Srour (France); Timm Harder, Anne Pohlmann and Christoph Staubach (Germany); Georgina Helyes and Malik Peter (Hungary); Brigitte Brugger and Vilhjálmur Svansson (Iceland); Laura Garza Cuartero, Audrey Jenkinson and Lucía Pérez (Ireland); Lara Cavicchio, Marta Dianati, Isabella Monne, Angela Salomoni and Bianca Zecchin (Italy); Juris Kibilds, Marta Lizuma and Žanete Šteingolde (Latvia); Paulius Bušauskas, Vilija Grigaliūnienė, Simona Pileviciene and Egidijus Pumputis (Lithuania); Guillaume Fournier and Chantal Snoeck (Luxembourg); Oxana Groza, Oxana Popa (Moldova); Monika Ballmann, Dennis Bol and Marcel Spierenburg (Netherlands); Aleksandar Dodovski and Igor Dzadzovski (North Macedonia); Anne Døsen and Britt Gjerset (Norway); Katarzyna Domańska-Blicharz, Anna Pikula and Magdalena Roman (Poland); Renata Carvalho, Margarida Duarte, Teresa Fagulha and Margarida Henriques (Portugal); Iuliana Onita, Radu Petrescu (Romania); Dejan Vidanovic (Serbia); Zuzana Dirbakova, Barbora Pavlíková and Martin Tinak (Slovakia); Brigita Slavec (Slovenia); Montserrat Agüero García, María Teresa Barrios Zambrano, Gérman Cáceres Garrido, Elena García Villacieros and Isabel María Guijarro Torvisco (Spain); Linnea Lindgren Kero and Siamak Zohari (Sweden); Claudia Bachofen and Julien Casaubon (Switzerland); Beverley McGarry, Michael McMenemy and Michelle Thompson (Northern Ireland)[1]). The authors also wish to thank Ron Fouchier from Erasmus Medical Center (Netherlands); Ashley Banyard and Holly Coombes from the Animal and Plant Health Agency (United Kingdom); Camille Delavenne from EpiMundi for conducting the data analysis under the contract EFSA/2025/OP/0014; the authors, originating and submitting laboratories of the sequences from GISAID's EpiFlu™ Database, which is used for this assessment (Annex D); Julio Álvarez and Jan Arend Stegeman from EFSA's Panel on Animal Health and Welfare (AHAW); Edoardo Colzani from ECDC, as well as Miguel Melo and Stefania Tampach from EFSA for the support provided to this scientific output.

[1] In accordance with the Agreement on the Withdrawal of the United Kingdom from the EU, and in particular with the Windsor Framework, the EU requirements on data sampling are also applicable to the United Kingdom (Northern Ireland).

Influenza), Barbezange, C., Buczkowski, H., Ducatez, M., Fusaro, A., Gonzalez, J. L., Kuiken, T., Mirinavičiūtė, G., Ståhl, K., Staubach, S., Svartström, O., Terregino, C., Willgert, K., Alarcón, A., & Kohnle, L. (2026). Avian influenza overview December 2025–February 2026. *EFSA Journal*, *24*(3), e10015, https://doi.org/10.2903/j.efsa.2026.10015

**ISSN:** 1831-4732

Reproduction is authorised provided the source is acknowledged.

EFSA may include images or other content for which it does not hold copyright. In such cases, EFSA indicates the copyright holder and users should seek permission to reproduce the content from the original source.

**Map disclaimer:** The designations employed and the presentation of material on any maps included in this scientific output do not imply the expression of any opinion whatsoever on the part of the European Food Safety Authority concerning the legal status of any country, territory, city or area or of its authorities, or concerning the delimitation of its frontiers or boundaries.

## Table of contents

Abstract ........ 1
Table of contents ........ 4
1 Introduction ........ 5
2 Assessment ........ 5
2.1 HPAI virus detections in birds ........ 5
2.1.1 HPAI virus detections in birds in Europe ........ 5
2.1.2 HPAI virus detections in birds outside Europe ........ 21
2.1.3 Genetic characteristics of HPAI viruses of the A(H5Nx) subtype in avian species ........ 25
2.2 HPAI virus detections in non-human mammals ........ 28
2.2.1 Genetic characteristics of HPAI viruses of the A(H5Nx) subtype circulating in Europe in non-human mammals ........ 34
2.3 Avian influenza virus infections in humans ........ 34
2.3.1 Overview of the most recent human infections with avian influenza viruses ........ 34
2.3.2 Human A(H5N1) cases ........ 35
2.3.3 Human A(H9N2) cases ........ 36
2.3.4 Human A(H10N3) cases ........ 37
2.3.5 Additional information relevant for public health and international risk assessments 37
2.3.6 ECDC risk assessment ........ 39
3 Conclusions ........ 40
3.1 Birds ........ 40
3.2 Mammals ........ 41
3.3 Humans ........ 42
4 Options for response ........ 42
4.1 Birds ........ 42
4.2 Mammals ........ 43
4.3 Humans ........ 45
References ........ 47
Appendix A – Terms of Reference of the joint EFSA-ECDC mandate (M-2024-00009) accepted in March 2024 ........ 59
Appendix B – Data and Methodologies ........ 61
Annex A – Data on HPAI virus detections in wild birds ........ 63
Annex B – Characteristics of the HPAI-affected poultry establishments ........ 63
Annex C – Control measures applied in Hungary to reduce secondary spread ........ 63
Annex D – Acknowledgements ........ 63

## 1 Introduction

This Scientific Report provides an overview of highly pathogenic avian influenza (HPAI) virus detections in poultry[2] and captive birds[3] (domestic birds), as well as in wild birds, that occurred in and outside Europe between 29 November 2025 and 27 February 2026. In addition, HPAI virus detections in mammals up until 13 February 2026 and cases of avian influenza infection in humans between 29 November 2025 and 27 February 2026 are reported. Detections of low pathogenic avian influenza (LPAI) virus in birds are discussed whenever they are of zoonotic concern or otherwise relevant.

The background, Terms of Reference (TOR), and interpretation thereof are described in Appendix A, whereas the data and methodologies used are reported in Appendix B.

## 2 Assessment

### 2.1 HPAI virus detections in birds

#### 2.1.1 HPAI virus detections in birds in Europe

Figure 1 shows all HPAI virus detections in birds in Europe that were reported via the European Union (EU) Animal Disease Information System (ADIS) or the World Animal Health Information System (WOAH-WAHIS) of the World Organisation for Animal Health (WOAH) for the last six and the current epidemiological year[4] by month of suspicion. For the current epidemiological year 2025–2026, starting on 1 October 2025, data reported are truncated on 27 February 2026.

[2] According to Regulation (EU) 2016/429 of the European Parliament and of the Council of 9 March 2016, point (9), 'poultry' means birds that are reared or kept in captivity for: (a) the production of: (i) meat; (ii) eggs for consumption; (iii) other products; (b) restocking supplies of game birds; (c) the purpose of breeding of birds used for the types of production referred to in points (a) and (b).
[3] According to Regulation (EU) 2016/429 of the European Parliament and of the Council of 9 March 2016, point (10), 'captive birds' means any birds other than poultry that are kept in captivity for any reason other than those referred to in point (9), including those that are kept for shows, races, exhibitions, competitions, breeding or selling.
[4] In this document an 'epidemiological year' refers to the period starting on 1 October and ending on 30 September of the following year, based on the dates on which the first HPAI virus detections were observed in wild birds in Europe in 2016–2017, 2020–2021 and 2021–2022.

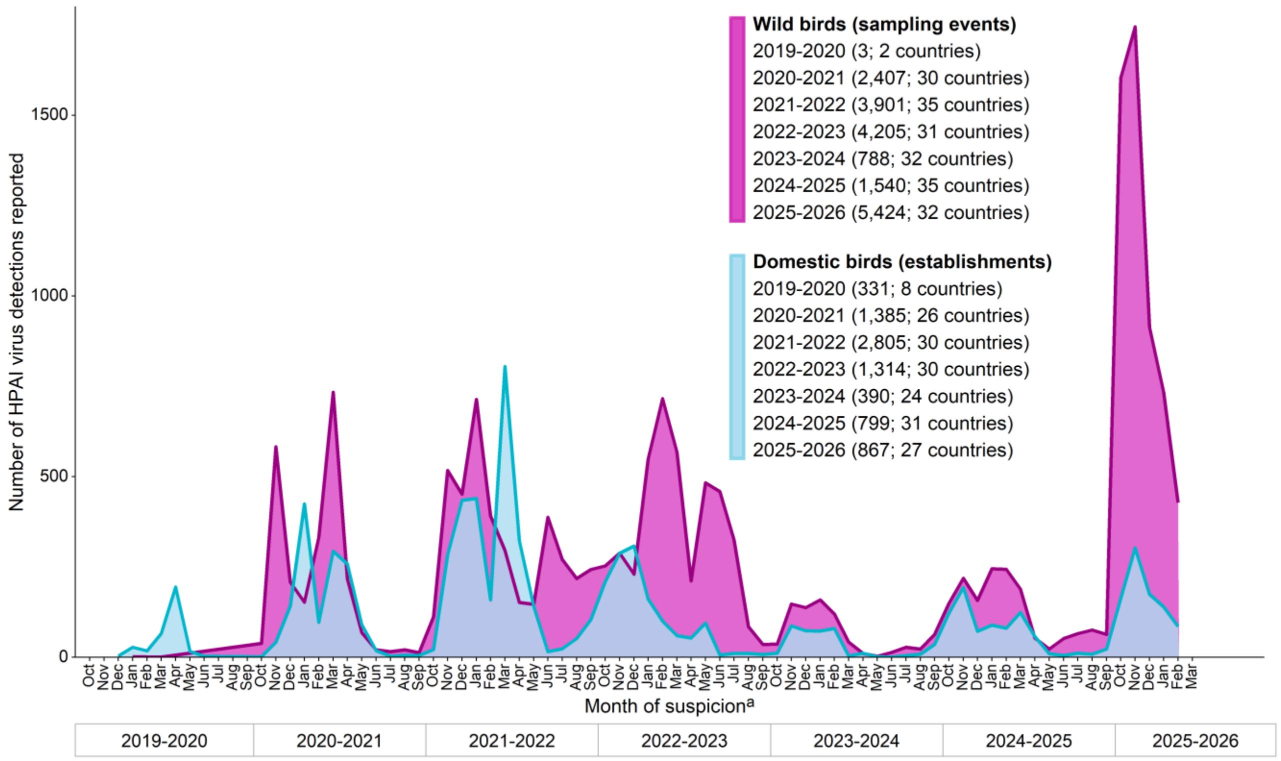

Figure 1: Distribution of the number of HPAI virus detections in wild birds (cumulative number n = 18,268) (pink) and establishments keeping domestic birds (cumulative n = 7891) (blue) reported in Europe during seven epidemiological years by month of suspicion, from 1 October 2019 to 27 February 2026 (total n = 26,159).

*Note*: United Kingdom data are from the Animal Disease Notification System (ADNS, former ADIS) up until 31 December 2020. From 1 January 2021 onwards, the data source was WOAH-WAHIS for the United Kingdom (excluding Northern Ireland) and ADNS/ADIS for the United Kingdom (Northern Ireland)[5].

[a] If the date of suspicion was not available, the date of confirmation was used to assign the week of suspicion.

*Source*: ADNS/ADIS and WOAH-WAHIS (data extraction carried out on 27 February 2026).

Considering the current reporting period from 29 November 2025 to 27 February 2026, 2514 HPAI virus detections were reported in poultry (289), captive (117) and wild (2108) birds in 32 countries in Europe (Table 1, Figure 2).

[5] In accordance with the Agreement on the Withdrawal of the United Kingdom from the EU, and in particular with the Windsor Framework, the EU requirements on data sampling are also applicable to the United Kingdom (Northern Ireland).

Table 1: Number of HPAI outbreaks reported in Europe by country, virus subtype and affected sub-population, from 29 November 2025 to 27 February 2026. Cumulative numbers since the start of the 2025-2026 epidemiological year are reported in parentheses (1 October 2025 to 27 February 2026).

| Country | Captive birds | Poultry | | | Wild birds | | | | Total |
|---|---|---|---|---|---|---|---|---|---|
| | A(H5N1) | A(Not typed) | A(H5Nx) | A(H5N1) | A(H5Nx) | A(H5N1) | A(H5N2) | A(H5N5) | |
| Austria | 2 (4) | - | - | 0 (1) | - | 29 (59) | - | - | 31 (64) |
| Belgium | 1 (3) | - | - | 11 (20) | 21 (37) | 159 (294) | - | - | 192 (354) |
| Bosnia and Herzegovina | - | 1 (1) | - | - | - | 1 (1) | - | - | 2 (2) |
| Bulgaria | 0 (1) | - | - | 3 (8) | - | - | - | - | 3 (9) |
| Croatia | - | - | - | - | - | 6 (6) | - | - | 6 (6) |
| Czechia | 20 (28) | - | - | 4 (13) | - | 33 (37) | - | - | 57 (78) |
| Denmark | 3 (4) | - | - | 8 (16) | - | 83 (134) | - | - | 94 (154) |
| Estonia | 3 (3) | - | - | - | - | 9 (9) | - | - | 12 (12) |
| Finland | - | - | - | - | 0 (1) | 7 (26) | - | - | 7 (27) |
| France | 18 (27) | - | - | 39 (118) | - | 53 (300) | - | - | 110 (445) |
| Germany | 38 (55) | - | - | 51 (189) | - | 852 (2914) | - | - | 941 (3158) |
| Hungary | - | - | - | 11 (13) | - | 40 (50) | - | - | 51 (63) |
| Iceland | - | - | - | - | 0 (1) | 2 (2) | - | 0 (1) | 2 (4) |
| Ireland | 0 (1) | - | - | 0 (5) | - | 3 (11) | - | - | 3 (17) |
| Italy | 2 (2) | - | - | 37 (62) | 11 (13) | 24 (91) | - | - | 74 (168) |
| Latvia | 1 (2) | - | - | - | - | 6 (26) | 0 (1) | - | 7 (29) |
| Lithuania | - | - | - | 1 (3) | - | 15 (25) | - | - | 16 (28) |
| Luxembourg | 1 (1) | - | - | - | - | 2 (23) | - | - | 3 (24) |
| Moldova | - | - | - | - | - | 5 (5) | - | - | 5 (5) |
| Netherlands | 7 (14) | - | - | 22 (39) | - | 112 (308) | - | - | 141 (361) |
| North Macedonia | 0 (2) | - | - | - | - | - | - | - | 0 (2) |
| Norway | - | - | - | - | 0 (9) | 29 (35) | - | - | 29 (44) |
| Poland | 9 (13) | - | - | 59 (79) | - | 135 (147) | - | - | 203 (239) |
| Portugal | 0 (4) | - | 0 (1) | 8 (11) | - | 4 (10) | - | - | 12 (26) |
| Romania | 1 (1) | - | - | 1 (1) | - | 7 (9) | - | - | 9 (11) |
| Serbia | - | - | - | - | - | 1 (1) | - | - | 1 (1) |
| Slovakia | 2 (4) | - | - | 3 (4) | - | 6 (9) | - | - | 11 (17) |
| Slovenia | - | - | - | - | - | 11 (17) | - | - | 11 (17) |
| Spain | 0 (4) | - | - | 2 (7) | 0 (1) | 27 (111) | - | - | 29 (123) |
| Sweden | 1 (2) | - | - | 3 (6) | - | 50 (73) | 1 (1) | - | 55 (82) |
| Switzerland | 0 (1) | - | - | - | - | 19 (24) | - | - | 19 (25) |
| Ukraine | 1 (1) | - | - | - | - | 1 (2) | - | - | 2 (3) |
| United Kingdom (excluding Northern Ireland) | 7 (18) | - | - | 25 (72) | - | 344 (591) | - | 0 (3) | 376 (684) |
| United Kingdom (Northern Ireland)[a] | - | - | - | 0 (3) | - | 0 (6) | - | - | 0 (9) |
| Total | **117 (195)** | **1 (1)** | **0 (1)** | **288 (670)** | **32 (62)** | **2075 (5356)** | **1 (2)** | **0 (4)** | **2514 (6291)** |

–, no HPAI outbreaks were notified via ADIS.

[a] In accordance with the Agreement on the Withdrawal of the United Kingdom from the EU, and in particular with the Windsor Framework, the EU requirements on data sampling are also applicable to the United Kingdom (Northern Ireland).

*Source*: ADIS and WOAH-WAHIS (data extraction carried out on 27 February 2026).

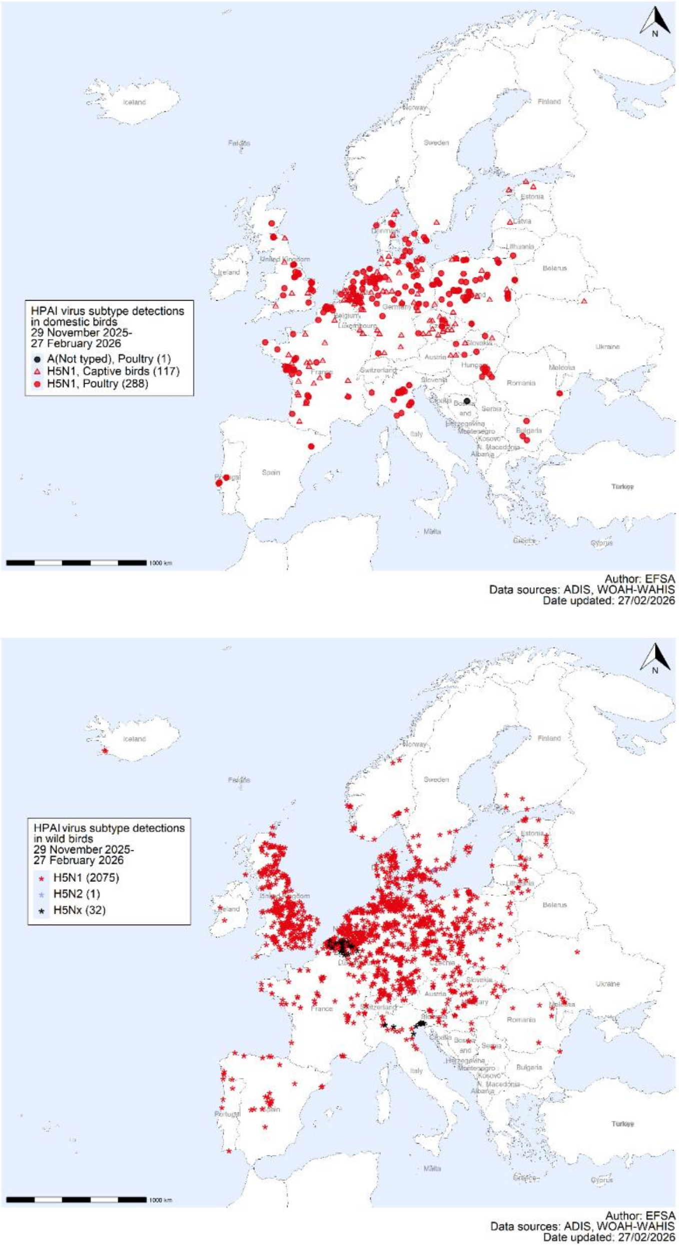

Figure 2: Geographic distribution, based on available geocoordinates, of HPAI virus detections in poultry and captive birds (406) (upper panel), and in wild birds (2108) (lower panel), reported by virus subtype in Europe from 29 November 2025 to 27 February 2026.

*Note*: United Kingdom (excluding Northern Ireland) data are from WOAH-WAHIS.

* This designation is without prejudice to positions on status and is in line with United Nations Security Council Resolution 1244 and the International Court of Justice Opinion on the Kosovo Declaration of Independence.

*Source*: ADIS and WOAH-WAHIS (data extraction carried out on 27 February 2026).

After a steep increase and sharp peak at the beginning of the current reporting period (end of November 2025), there was a steady decline in the number of HPAI virus detections in both wild and domestic birds throughout the following weeks up to the end of the current reporting period (end of February 2026) (Figure 1, Table 1). The number of detections in domestic birds in the current reporting period was similar to those in the past two years, while the number of detections in wild birds was three times higher than in the same time period last year, and almost five times higher than in the same time period two years ago. This was particularly the case for detections in waterfowl, which continued to show a two-peak behaviour as in previous years (Figure 3).

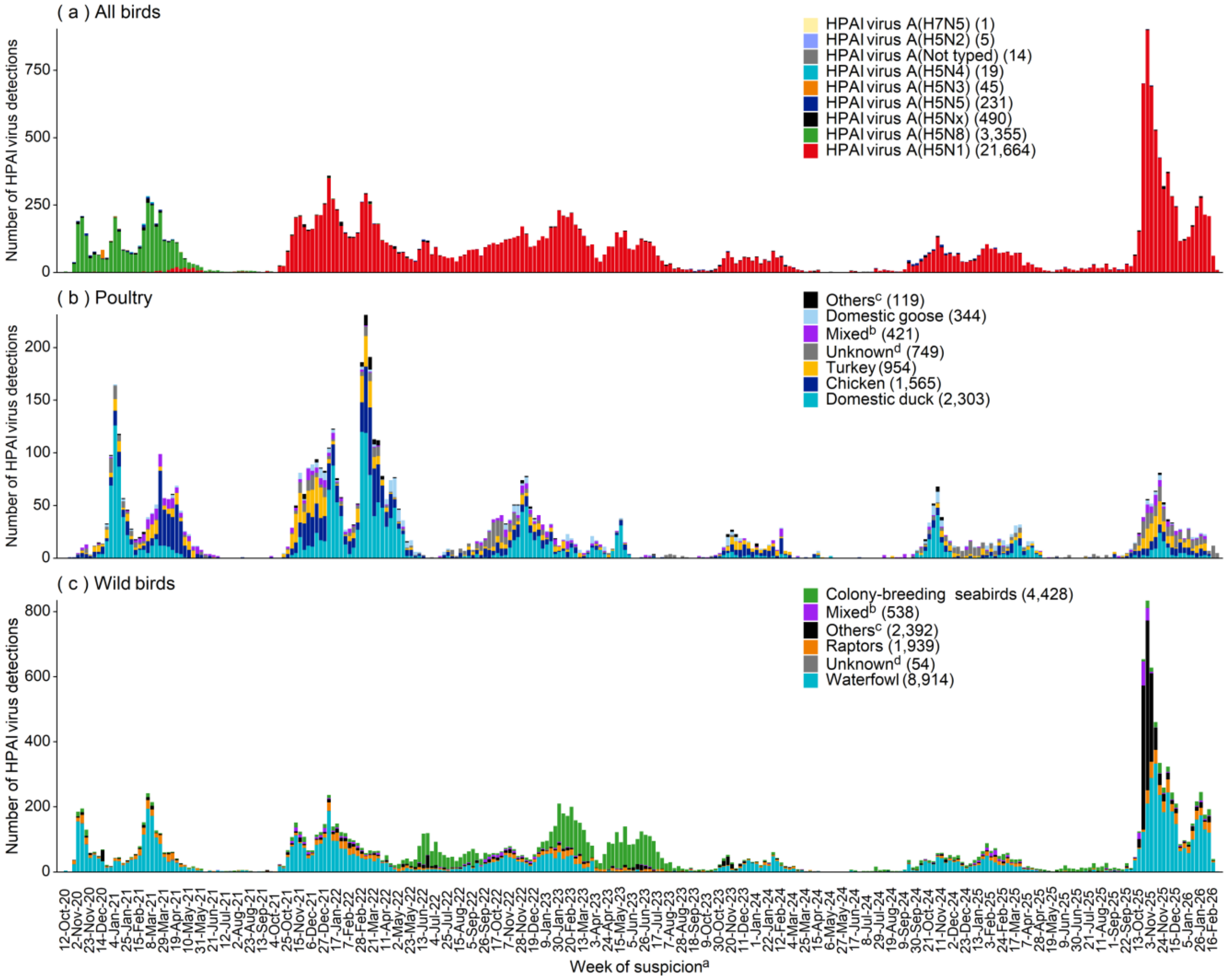

Figure 3: Distribution of the total number of HPAI virus detections reported in Europe by week of suspicion (dates indicate the first day of the week) and virus subtype (a), affected poultry categories (b) and affected wild bird categories (c), from 1 October 2020 to 27 February 2026.

[a] If the date of suspicion was not available, the date of confirmation was used to assign the week of suspicion.

[b] Mixed, outbreaks in which multiple species or categories were involved.

[c] Others, all other affected categories that are not indicated in the legend.

[d] Unknown, affected categories that were not further specified during reporting.

*Source*: ADNS/ADIS, EFSA and WOAH-WAHIS (data extraction carried out on 27 February 2025).

The temporal and geographical distributions of HPAI outbreaks in domestic birds (Figure 1; Figure 2, upper panel) largely overlapped with those in wild birds (Figure 2, lower panel; Figure 3). In the current reporting period, HPAI A(H5N1) virus detections in both domestic and wild birds were concentrated in the central–western part of Europe, roughly from Poland in the east to the United Kingdom (excluding Northern Ireland) in the west, as well as in the north of Italy (Figure 2). Relatively few HPAI virus detections in domestic birds compared to wild birds were reported in the United Kingdom. In contrast to the previous reporting period, when a few HPAI A(H5N5) virus detections were reported from Iceland, the United Kingdom (excluding Northern Ireland), and Norway, there were none in the current reporting period. However, in addition to the data available via ADIS, the Danish national reference laboratory reported the detection of the HPAI A(H5N5) genotype EA-2021-I in a black-headed gull found dead in January in Denmark.

Spatio-temporal information on all HPAI virus detections reported in Europe since October 2016 is available via EFSA's interactive dashboard[6].

### Poultry

Between 29 November 2025 and 27 February 2026, 289 HPAI outbreaks in poultry were reported from 18 countries in Europe: Poland (59), Germany (51), France (39), Italy (37), United Kingdom (excluding Northern Ireland) (25), Netherlands (22), Belgium (11), Hungary (11), Denmark (8), Portugal (8), Czechia (4), Bulgaria (3), Slovakia (3), Sweden (3), Spain (2), Bosnia and Herzegovina (1), Lithuania (1), and Romania (1) (Table 1, Figure 2, Figure 4).

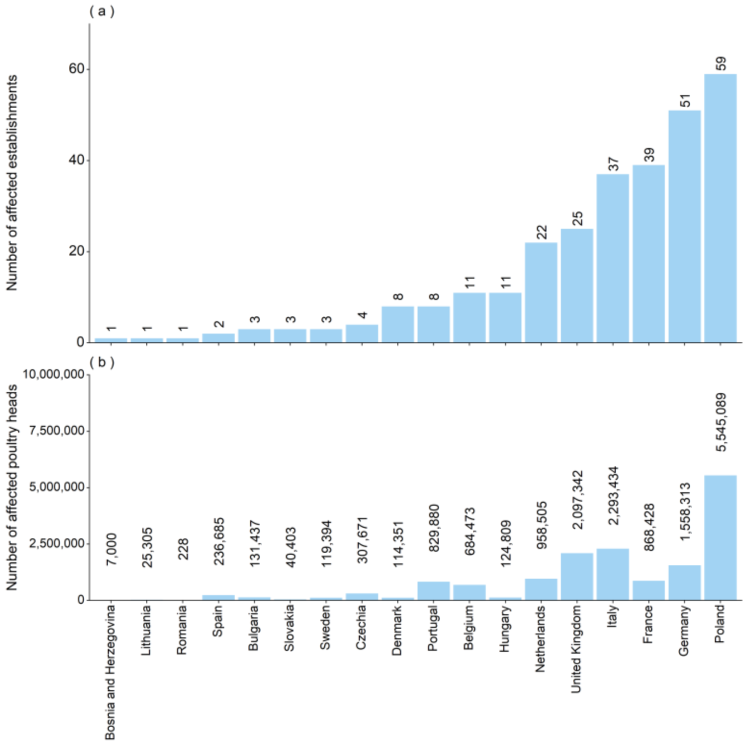

Figure 4: Number of HPAI-affected establishments (289) (a) and number of poultry heads in the HPAI-affected establishments (15,942,747) (b) per country in Europe between 29 November 2025 and 27 February 2026 (countries are ranked according to the number of outbreaks).

[6] http://hpai.efsa.aus.vet/

*Note*: United Kingdom (excluding Northern Ireland) data are from WOAH-WAHIS.

*Source*: ADIS, EFSA and WOAH-WAHIS (data extraction carried out on 27 February 2026).

In the current reporting period, almost all (99.7%, 288/289) HPAI outbreaks in poultry were due to HPAI A(H5N1) viruses, while in one outbreak the subtype involved was not further identified (A(H5Nx), 0.3%, 1/289). This compares to a total of 167 outbreaks in the same period in the previous epidemiological year, among which 160 (95.8%) were due to HPAI A(H5N1), 6 (3.6%) were due to HPAI A(H5Nx), and one (0.6%) was due to HPAI A(H5N5) virus. In the current reporting period, Poland and Germany accounted for 38% (110/289) of the HPAI outbreaks in poultry and 45% (7,103,402/15,942,747) of the number of poultry heads culled (Figure 4). In total, almost 16 million birds died or were culled in the HPAI-affected establishments (Figure 4), about 27% more than in the previous reporting period. Overall, 82% (237/289) of the HPAI outbreaks in poultry were classified as primary, 9% (27/289) as secondary, and for 25 outbreaks (9%), this information was not available. Although the number of HPAI virus detections detected in waterfowl during the current epidemiological year (EFSA and EURL, 2025), and particularly during the current reporting period, is much higher than in previously characterised large epidemics (2020–2021, 2021–2022 and 2022–2023) (Figure 3), the number of HPAI outbreaks in poultry is lower. Different from those epidemiological years, most of the outbreaks in this reporting period were primary introductions, with only limited reported farm-to-farm transmission. Most of the secondary outbreaks reported in the current reporting period occurred in three countries: Belgium (9), Poland (7), and Portugal (7). Between 29 November 2025 and 27 February 2026, the most affected poultry category in Europe was the 'chicken' category (21.4%, 62/289 outbreaks), followed by the 'turkey' (19.4%, 56/289), 'domestic duck' (13.8%, 40/289), 'domestic goose' (3.5%, 10/289), and 'other' (1.4%, 4/289) categories. Multiple species were kept in 21 (7.3%) establishments, and no information was available on the species kept for another 96 (33.2%) establishments (Figure 3).

In the following paragraphs, a brief description of the HPAI outbreaks in poultry is given by country. This description is based on information collected by EFSA from ADIS and WOAH-WAHIS, reporting countries (in form of additional data submitted and personal communications), and media reports. In the period from 29 November 2026 to 27 February 2026, 289 HPAI outbreaks in poultry were reported in Europe via ADIS or WOAH-WAHIS. Additional data on the characteristics of the affected poultry establishments (e.g. poultry species, production type, source of introduction, number of exposed people, clinical signs and mortality) were collected for 193 of these 289 (67%) from Czechia, Denmark, France, Germany, Hungary, Lithuania, Netherlands, Poland, Portugal, Romania, Slovakia, Spain, and Sweden (Annex B). Among those, 175 (91%) were reported as primary and 18 (9%) as secondary. In 64% (112/175) of the primary outbreaks, indirect contact with wild birds was considered the most likely source of introduction, followed by direct contact with wild birds (1%, 1/175), and indirect contact with poultry (1%, 1/175). For the remaining 34% (60/175), the source was reported as unknown. In 39% (7/18) of secondary outbreaks, indirect contact with wild birds was considered the most likely source of introduction, followed by indirect contact with poultry (33%, 6/18). For the remaining 28% (5/18), the source was reported as unknown. For the other 96 (33%) outbreaks in poultry, no additional data were collected, either because countries did not submit those data or did not report them via ADIS, or because they occurred too close to the publication of this report. Therefore, only a short summary of those outbreaks will be provided here, while additional data will only be collected in the following round and included in Annex B of the following report.

*Belgium*

During the current reporting period from 29 November 2025 to 27 February 2026, eleven HPAI A(H5N1) outbreaks in poultry were reported in Belgium via ADIS. No additional data to complement the information in ADIS were provided by the country. Among these outbreaks, 82% (9/11) were classified as secondary and 18% (2/11) as primary. In total, 684,473 birds were affected across the eleven establishments. According to ADIS, most outbreaks occurred in establishments keeping broilers (55%, 6/11), followed by chickens for breeding (18%, 2/11), turkeys for fattening (18%, 2/11), and laying hens (9%, 1/11). In all establishments mortality (median: 0.25%, range 0.05–0.49%) was observed and clinical signs were reported.

*Bosnia and Herzegovina*

During the current reporting period from 29 November 2025 to 27 February 2026, one primary HPAI (subtype pending) outbreak in poultry was reported in Bosnia and Herzegovina via ADIS. The outbreak was reported on 24 February and involved 7,000 susceptible birds, with a reported mortality of 21.4% and clinical signs present. As the outbreak occurred after data collection had been closed on 13 February 2026, no further information was collected for the present report.

*Bulgaria*

During the current reporting period from 29 November 2025 to 27 February 2026, three primary HPAI A(H5N1) outbreaks in poultry were reported in Bulgaria via ADIS. No additional data to complement the information in ADIS were provided by the country. The three outbreaks affected a total of 131,437 birds and, according to ADIS, two outbreaks (67%) occurred in establishments keeping ducks (n = 15,237; n = 26,200), while one outbreak (33%) affected an establishment keeping laying hens (n = 90,000). In all establishments mortality (median: 4.19%, range 1.11–4.28%) was observed and clinical signs were reported.

*Czechia*

During the current reporting period from 29 November 2025 to 27 February 2026, four HPAI A(H5N1) outbreaks in poultry were reported in Czechia via ADIS. Additional data were collected and provided by the reporting country for three of these outbreaks (Annex B), while no further information was collected for the fourth outbreak, which occurred after 13 February, as data collection had already been closed. Among the three outbreaks with additional information available, 67% (2/3) were classified as primary and 33% (1/3) as secondary. Two of them occurred in commercial establishments keeping chickens for breeding (n = 30,000; n = 35,000), while one affected a commercial establishment keeping ducks for breeding (n = 6,789). Indirect contact with wild birds was identified as the most likely source of introduction in these three outbreaks. Overall, 307,671 birds were affected across the four establishments. Mortality (median: 6.84%; range: 0.15–14.7%) and clinical signs, including a drop in egg production in the ducks, were observed in all of them. A total of 175 persons were reported as exposed across the three establishments with additional information available.

*Denmark*

During the current reporting period from 29 November 2025 to 27 February 2026, eight primary HPAI A(H5N1) outbreaks in poultry were reported in Denmark via ADIS. Additional data were collected and provided by the reporting country for four of these outbreaks (Annex B), while no further information was collected for the other four outbreaks, which occurred after 13 February, as data collection had already been closed. Among the outbreaks with additional information available, 50% (2/4) occurred in commercial establishments keeping laying hens (n = 14,000)

and mixed species (pheasants, laying hens, ornamental birds) (n = 546), while another 50% (2/4) occurred in non-commercial establishments keeping mixed species (pigeons and chickens for mixed purposes, n = 26; laying hens and ducks, n = 19). Indirect contact with wild birds was identified as the most likely source of introduction in all four outbreaks. Overall, 114,351 birds were affected across the eight establishments. Mortality (median: 19.6%, range: 1.43–36.84%) was observed in all, while clinical signs, including depression and nasal discharge (European Commission, online), were only reported in two of them. A total of 40 people were reported as exposed in the four establishments with additional information available.

*France*

During the current reporting period from 29 November 2025 to 27 February 2026, 39 HPAI A(H5N1) outbreaks in poultry were reported in France via ADIS. Additional data were collected and provided by the reporting country for all outbreaks (Annex B). Among these, 97% (38/39) were classified as primary and 3% (1/39) as secondary, affecting a total of 868,428 birds. Most (87%, 34/39) of the outbreaks occurred in commercial single-species establishments, including vaccinated ducks (36%, 14/39), turkeys for fattening (15%, 6/39), laying hens (10%, 4/39), broilers (8%, 3/39), chickens for breeding (5%, 2/39), pheasants for breeding (5%, 2/39), geese for breeding (3%, 1/39), quails for breeding (3%, 1/39), and guineafowl for fattening (3%, 1/39), while the remaining 13% (5/39) of the outbreaks occurred in commercial mixed-species establishments. Indirect contact with wild birds was identified as the most likely source of introduction in 18% (7/39) of the outbreaks, followed by indirect contact with poultry (3%, 1/39), while no information was available for 79% (31/39). A general housing order was in place during the current reporting period. Clinical signs were reported in 87% (34/39) of the establishments, with no information available for the remaining 13% (5/39). Mortality was reported in 82% (32/39) of the outbreaks (median: 0.33%; range: 0.019–10.56%), while it was not observed in the remaining 18% (7/39). A total of 26 persons were reported as exposed in four of the outbreaks.

Among the outbreaks in vaccinated ducks, 93% (13/14) were classified as primary and 7% (1/14) as secondary. These outbreaks included ducks for foie gras production (57%, 8/14), ducks for fattening (29%, 4/14), ducks for breeding (7%, 1/14), and ducks for mixed purposes (7%, 1/14), affecting a total of 156,709 birds. None of the vaccinated ducks had outdoor access. Mortality and clinical signs were reported in 64.3% (9/14) of the outbreaks (median: 0.13%, range: 0.04–0.96%), of which 89% (8/9) were detected via passive and 11% (1/9) via active surveillance. Clinical signs without the presence of mortality were observed in two (14.3%, 2/14) outbreaks, which were detected via passive (50%, 1/2) and active (50%, 1/2) surveillance, respectively. Three (21.4%, 3/14) other outbreaks were not associated with mortality or clinical signs. These outbreaks were detected via enhanced passive (33%, 1/3), active (33%, 1/3), and outbreak-related (33%, 1/3) surveillance, respectively.

*Germany*

During the current reporting period from 29 November 2025 to 27 February 2026, 51 primary HPAI A(H5N1) outbreaks in poultry were reported in Germany via ADIS. Additional data were collected and provided by the reporting country for 48 of these outbreaks (Annex B), while no further information was collected for the other three outbreaks, which occurred after 13 February, as data collection had already been closed. Among the outbreaks with additional information available, 83% (40/48) occurred in commercial single-species establishments keeping turkeys for fattening (40%, 19/48), turkeys for breeding (13%, 6/48), chickens for breeding (8%, 4/48), broilers (8%, 4/48), laying hens (6%, 3/48), chickens for mixed purposes (4%, 2/48), ducks for fattening (2%, 1/48), and geese for breeding (2%, 1/48). Mixed-species establishments represented 17% (8/48) of these outbreaks, of which 75% (6/8) were commercial and 25% (2/8) non-commercial. Outdoor access was provided in 15% (7/48) of the establishments, while 85% (41/48) kept birds indoors. Indirect contact with wild birds was identified as the most likely source of introduction in all 48 outbreaks with additional information available. Overall, 1,558,313 birds were affected across the 51 affected establishments. Mortality was observed in 96% (46/48) of the outbreaks (median: 0.56%; range: 0.03–89.5%), while clinical signs were reported in 29% (14/48).

*Hungary*

During the current reporting period from 29 November 2025 to 27 February 2026, 11 primary HPAI A(H5N1) outbreaks in poultry were reported in Hungary via ADIS. Additional data were collected and provided by the reporting country for ten of these outbreaks (Annex B), while no further information was collected for the eleventh outbreak, which occurred after 13 February, as data collection had already been closed. In total, 124,809 birds were affected across the 11 affected establishments. The outbreaks with additional information available involved commercial establishments keeping geese for foie gras production (30%, 3/10), geese for breeding (20%, 2/10), turkeys for fattening (20%, 2/10), broilers (10%, 1/10), turkeys for breeding (10%, 1/10), and ducks for fattening (10%, 1/10). Outdoor access was reported in one (10%) establishment keeping geese for foie gras production, while 90% (9/10) of establishments did not provide outdoor access. Indirect contact with wild birds was identified as the most likely source of introduction in all outbreaks with additional information available. A housing order in high-risk areas as well as certain measures to reduce secondary spread (Annex C) were in place during the current reporting period. Mortality was observed in all these outbreaks (median: 11.3%; range: 0.39–52.3%). Clinical signs, including neurological signs, lethargy, and decreased feed and water intake, were reported in 50% (5/10) of the outbreaks (Nehib, online-a,b), while the remaining 50% (5/10) did not report any. A total of 119 persons were reported as exposed across the ten outbreaks with additional information available.

*Italy*

During the current reporting period from 29 November 2025 to 27 February 2026, 37 primary HPAI A(H5N1) outbreaks in poultry were reported in Italy via ADIS. No additional data to complement the information in ADIS were provided by the country. In total, 2,293,434 birds were affected, most of which were in large commercial establishments located in high-density poultry areas in northern parts of the country (European Commission, online). Phylogenetic analyses indicate close genetic relatedness among the viruses detected in the different outbreaks. However, these data do not allow inference of transmission dynamics or attribution of specific pathways. Epidemiological investigations and contact tracing conducted during the most recent outbreaks have not identified any evidence of secondary transmission associated with human activities. The potential role of wild bird species, not systematically included in surveillance programmes (e.g. sacred ibises and cattle egrets commonly present in rural areas) in the dissemination of closely related viruses from farm to farm cannot be excluded. Currently, however, there is no direct evidence to support this theory. In some investigations, shortcomings in the implementation of biosecurity measures were highlighted as potential contributing factors to disease spread. According to ADIS, the outbreaks involved establishments keeping turkeys for fattening (60%, 22/37), laying hens (32%, 12/37), geese (3%, 1/37), and mixed species (5%, 2/37). Mortality was reported in 97% (36/37) of the outbreaks (median: 0.20%; range: 0.008–100%), while clinical signs were observed in all. Mortality and presence of clinical signs varied according to species, production type and the stage at which the outbreak was detected. Increased mortality and/or sensory depression were commonly observed, together with reduced feed and water intake in lethargic flocks. In subacute cases, neurological (ataxia, incoordination), respiratory (gasping, cyanosis) and digestive (diarrhoea) signs were observed. Post-mortem examinations revealed multifocal punctiform haemorrhages affecting various organs, including air sacs and visceral and epicardial fat, as well as splenomegaly with diffuse necrotic foci.

*Lithuania*

During the current reporting period from 29 November 2025 to 27 February 2026, one secondary HPAI A(H5N1) outbreak in poultry was reported in Lithuania via ADIS, for which additional data were collected and provided by the reporting country (Annex B). The outbreak was reported on 5 December in a commercial establishment keeping turkeys for fattening (n = 25,305), where mortality of 0.34% was observed but no clinical signs were reported. The establishment was in close proximity to and under the same ownership as another previously affected establishment. A general housing order was in place during the current reporting period. Twenty-four persons were reported as exposed.

*Netherlands*

During the current reporting period, from 29 November 2025 to 27 February 2026, 22 primary HPAI A(H5N1) outbreaks in poultry were reported in the Netherlands via ADIS. Additional data were collected and provided by the reporting country for 18 of these outbreaks (Annex B), while no further information was collected for the other four, which occurred after 13 February, as data collection had already been closed. In total, 958,505 birds were affected across the 22 establishments. The outbreaks with additional information available involved commercial establishments keeping laying hens (33%, 6/18), broilers (28%, 5/18), ducks for fattening (22%, 4/18), and turkeys for fattening (17%, 3/18). Mortality was reported in 67% (12/18) of these outbreaks (median: 0.05%; range: 0.01–0.61%), while it was not observed in 33% (6/18). In these cases, HPAI was detected at an early stage on the basis of a slight increase in mortality or other clinical and laboratory indicators. Clinical signs were reported in all 18 outbreaks, including lethargy, reddened combs, drop in production, and reduced feed intake. Pathological findings such as petechiae in the heart and kidneys were also observed (European Commission, online).

*Poland*

During the current reporting period from 29 November 2025 to 27 February 2026, 59 HPAI A(H5N1) outbreaks in poultry were reported in Poland via ADIS. Additional data were collected and provided by the reporting country for 54 of these outbreaks (Annex B), while no further information was collected for the other five, which occurred after 13 February, as data collection had already been closed. In total, 5,545,089 birds were affected across the 59 establishments. The majority of outbreaks occurred in the Wielkopolskie Voivodeship, a high-density poultry area. Among the outbreaks with additional information available, 87% (47/54) were classified as primary and 13% (7/54) as secondary. These outbreaks involved commercial establishments keeping ducks for fattening (30%, 16/54), turkeys for fattening (28%, 15/54), laying hens (17%, 9/54), broilers (7%, 4/54), chickens for breeding (7%, 4/54), geese for breeding (6%, 3/54), ducks for breeding (2%, 1/54), and mixed species (4%, 2/54). Outdoor access was reported in 24% (13/54) of establishments, while 76% (41/54) did not provide outdoor access. Indirect contact with wild birds was identified as the most likely source of introduction in 69% (37/54) of the outbreaks, followed by indirect contact with poultry (11%, 6/54), while no information on the source was available for 20% (11/54). Local housing orders were in place during the current reporting period. Mortality was reported in all outbreaks (median: 1.14%; range: 0.007–100%), while clinical signs were observed in 83% (45/54). Among the 54 outbreaks with additional information available, the total number of exposed persons was 1,742.

*Portugal*

During the current reporting period from 29 November 2025 to 27 February 2026, eight HPAI A(H5N1) outbreaks in poultry were reported in Portugal via ADIS. Additional data were collected and provided by the reporting country for all these outbreaks (Annex B). Among these, 87.5% (7/8) were classified as secondary and 12.5% (1/8) as primary. Most of the secondary outbreaks occurred in close proximity to one another and in already restricted areas, while in one outbreak personnel were shared with another affected establishment of the same company. In total, 829,880 birds were affected across the eight establishments. All outbreaks occurred in commercial establishments keeping ducks for fattening (25%, 2/8), turkeys for fattening (25%, 2/8), chickens for breeding (12.5%, 1/8), laying hens (25%, 2/8), and broilers (12.5%, 1/8). Indirect contact with wild birds was identified as the most likely source of introduction in 62.5% (5/8) of the outbreaks, while no information on the source was available for the remaining 37.5% (3/8). A general housing order was in place up to 16 February 2026. After that, it was restricted to high-risk areas. Mortality was observed in all outbreaks (median: 0.32%; range: 0.15–4.78%), while clinical signs were observed in 62.5% (5/8). All outbreaks included exposed persons, comprising a total of 271 individuals.

*Romania*

During the current reporting period from 29 November 2025 to 27 February 2026, one primary HPAI A(H5N1) outbreak in poultry was reported in Romania via ADIS, for which additional data were collected and provided by the reporting country (Annex B). The outbreak was reported on 3 February in a non-commercial establishment keeping mixed species (turkeys, ducks, geese, chickens) (n = 228) with outdoor access. Direct contact with wild birds was identified as the most likely source of introduction. Mortality of 26.31% was observed but no clinical signs were reported. A housing order was implemented in the protection and surveillance zones during the current reporting period. Two persons were reported as exposed.

*Slovakia*

During the current reporting period, from 29 November 2025 to 27 February 2026, three primary HPAI A(H5N1) outbreaks in poultry were reported in Slovakia via ADIS. Additional data were collected and provided by the reporting country for two of these outbreaks (Annex B), while no further information was collected for the other one, which occurred after 13 February, as data collection had already been closed. In total, 40,403 birds were affected across the three affected establishments. The outbreaks with additional information available involved two commercial establishments keeping turkeys for fattening (n = 1305) and mixed species (n = 36,000). The latter included turkeys for fattening, laying hens and pullets, which had outdoor access. Indirect contact with wild birds was identified as the most likely source of introduction in this outbreak, while no information was available for the other. In both outbreaks mortality (median: 18.05%; range: 0.61–41.9%) and clinical signs, including decreased feed intake, decreased laying rate, and signs consistent with HPAI, were reported (European Commission, online; Regióny.sk, online).

*Spain*

During the current reporting period from 29 November 2025 to 27 February 2026, two HPAI A(H5N1) outbreaks in poultry were reported in Spain via ADIS. Additional data were collected and provided by the reporting country for both outbreaks (Annex B), one of which was identified as primary and one as secondary. The primary outbreak was reported on 22 December in a commercial establishment keeping laying hens (n = 227,074), where mortality of 0.35% and clinical signs were observed. Indirect contact with wild birds was identified as the most likely source of introduction (European Commission, online). The secondary outbreak was reported on 8 January in a commercial establishment keeping chickens for breeding (n = 9,611), located in proximity to the primary outbreak, with mortality of 1.92% and clinical signs reported. A general housing order was in place during the current reporting period. Fourteen persons were reported as exposed in the latter.

*Sweden*

During the current reporting period from 29 November 2025 to 27 February 2026, three primary HPAI A(H5N1) outbreaks in poultry were reported in Sweden via ADIS. Additional data were collected and provided by the reporting country for all these outbreaks (Annex B). In total, 119,394 birds were affected across the three establishments. All outbreaks occurred in commercial establishments keeping chickens for breeding without clinical signs, but mortality was observed in all establishments (median: 0.3%, range: 0.19–0.50%). Indirect contact with wild birds was identified as the most likely source of infection for all outbreaks, and a total of 43 persons were reported as exposed. A general housing order was in place in the areas where these outbreaks occurred during the current reporting period.

*United Kingdom (excluding Northern Ireland)*

During the current reporting period from 29 November 2025 to 27 February 2026, 25 A(H5N1) outbreaks in poultry were reported in the United Kingdom (excluding Northern Ireland) via WOAH-WAHIS. In total, 2,097,342 birds were affected. Among the 25 establishments, 24% (6/25) kept laying hens, 20% (5/25) turkeys for fattening, 12% (3/25) chickens for breeding, 8% (2/25) pullets, 4% (1/25) broilers, 4% (1/25) ducks for breeding, and 4% (1/25) ducks for fattening. Another 8% (2/25) kept mixed species, and this information was not available for four (16%) outbreaks. Mortality was reported in all establishments (median: 0.39%; range: 0.02–19.71%), while no information on clinical signs was available.

**Captive birds**

Between 29 November 2025 and 27 February 2026, 117 HPAI outbreaks in captive birds were reported from 18 countries in Europe: Germany (38), Czechia (20), France (18), Poland (9), Netherlands (7), United Kingdom (excluding Northern Ireland) (7), Denmark (3), Estonia (3), Austria (2), Italy (2), Slovakia (2), Belgium (1), Latvia (1), Luxembourg (1), Romania (1), Sweden (1), and Ukraine (1) (Table 1, Figure 2).

Most outbreaks occurred in non-commercial or backyard establishments keeping small numbers of mixed poultry species for private consumption, particularly in Czechia, Poland, Latvia, and Denmark. Many of these hobby flocks were composed of laying hens, ducks, geese and pigeons that were kept together. Sudden death and clinical signs were frequently reported in these outbreaks, and in some cases, mass mortality was observed.

In addition, outbreaks were confirmed in establishments open to the public, including zoos, petting farms and animal sanctuaries. In Czechia, HPAI A(H5N1) was reported at Prague Zoo on

12 February 2026 following increased mortality in birds kept in the Pelican Exhibit and the Asian Lagoon, including pelicans and other waterbirds. Eight birds died, while 87 showed no clinical signs (European Commission, online; Metro Czechia, online). Similar events were reported by other countries. In Denmark, HPAI was reported in an ibis at Odense Zoo (Fyens, online). In Germany, several zoological parks reported cases, such as a nandu at Osnabrück Zoo, ducks and geese in a separate seabird aviary at Weltvogelpark Walsrode, Dalmatian pelicans at Leipzig Zoo, and geese and ducks at Chemnitz Zoo (Diezeit, online; Kieler Nachrichten; NDR, online; online; Volksstimme, online). According to ADIS, another zoo was affected in France. In the Netherlands, outbreaks involved small animal zoos and petting farms, including captive chickens and other ornamental birds (Netherlands Food and Consumer Product Safety Authority, online; RTV Utrecht, online). Additional outbreaks in establishments exhibiting animals to the public were reported in Slovakia, involving birds of prey kept in captivity (Spravy, online), and in Sweden, where a school collection of ducks and other bird species was affected.

**Wild birds**

During the current reporting period from 29 November 2025 to 27 February 2026, a total of 2108 HPAI virus detections in wild birds (with one HPAI virus detection potentially including more than one wild bird species) were reported from Germany (852), United Kingdom (excluding Northern Ireland) (344), Belgium (180), Poland (135), Netherlands (112), Denmark (83), France (53), Sweden (51), Hungary (40), Italy (35), Czechia (33), Austria (29), Norway (29), Spain (27), Switzerland (19), Lithuania (15), Slovenia (11), Estonia (9), Finland (7), Romania (7), Croatia (6), Latvia (6), Slovakia (6), Moldova (5), Portugal (4), Ireland (3), Iceland (2), Luxembourg (2), Bosnia and Herzegovina (1), Serbia (1) and Ukraine (1).

Overall, 2075 HPAI virus detections in wild birds were reported as A(H5N1), 32 as A(H5Nx) and one as A(H5N2) (Table 1, Figure 2). The overall number of HPAI virus detections reported in wild birds (2108) in the current reporting period was about a third lower than in the previous reporting period (3370) and three times higher than in the same period of the previous epidemiological year (660) (Figure 3). Regarding the wild bird categories involved and considering that more than one wild bird species can be included in a single HPAI virus detection, HPAI A(H5) was mostly recorded in waterfowl (72%, 1510/2108), followed by raptors (13%, 284/2108), colony-breeding seabirds (7%, 157/2108), and 'other' wild bird species (6%, 130/2108) (Figure 5).

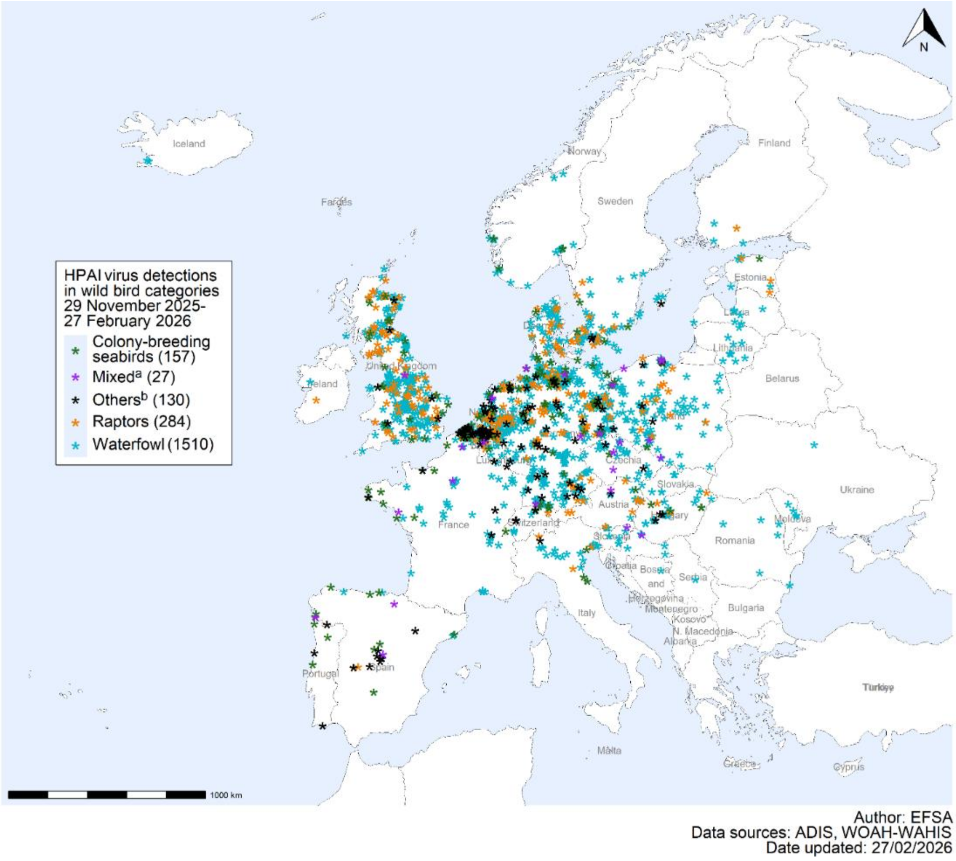

Figure 5: Geographic distribution, based on available geocoordinates, of HPAI virus detections in different categories of wild birds in Europe, by species category, from 29 November 2025 to 27 February 2026.

*Notes*: the unit reported is the number of HPAI virus detections in different wild bird categories and not the total number of HPAI virus detections in wild birds (as more than one species can be involved in one single HPAI virus detection reported).

United Kingdom (excluding Northern Ireland) data are from WOAH-WAHIS.

[a] Mixed, outbreaks in which multiple species or categories were involved.
[b] Others, all other affected categories that are not indicated in the legend.
[c] Unknown, outbreaks for which no information on the wild bird species involved is available.

* This designation is without prejudice to positions on status and is in line with United Nations Security Council Resolution 1244 and the International Court of Justice Opinion on the Kosovo Declaration of Independence.

*Source*: ADIS and WOAH-WAHIS (data extraction carried out on 27 February 2026).

This pattern differs from the previous reporting period, when other birds, mainly common cranes, constituted a substantial proportion (41%) of HPAI virus detections in wild birds, compared to 6% in the current reporting period. This abrupt decline in HPAI virus detections in common cranes corresponds to the end of their autumn migration. There were only four HPAI virus detections in common cranes in the current reporting period. In contrast, the proportions of detections in waterfowl (72% vs 44%) and raptors (13% vs 6%) increased in the current reporting period compared to the previous one. Notably, the number of HPAI virus detections in waterfowl in the current reporting period (1510) was four times higher than in the same period in the 2024–2025 epidemiological year (354 HPAI virus detections in waterfowl), and five times higher than in the 2023–2024 epidemiological year (289 HPAI virus detections in waterfowl) (Figure 3). This is likely correlated to the higher circulation of HPAI virus in the waterfowl population during the current epidemiological year. The geographical distribution of HPAI virus detections in raptors and colony-breeding seabirds largely overlapped with those in waterfowl, which were concentrated in the central-western part of Europe, where many waterfowl spend the winter.

The wild bird species in which HPAI viruses were detected belonged mainly to four orders: Anseriformes (1583), Accipitriformes (241), Charadriiformes (168), and Pelecaniformes (49). The main Anseriformes identified to species were the mute swan (425 vs 244 in the previous reporting period), greylag goose (216 vs 149), Canada goose (142 vs 108), and barnacle goose (101 vs 26) (Figures A.1–A.2 in Annex A). In addition, there were 373 unidentified Anatidae. The main Accipitriformes identified to species were the Eurasian buzzard (158 vs 63), Eurasian sparrowhawk (12 vs 3), and Northern goshawk (9 vs 3). In addition, there were 52 unidentified Accipitridae. The main Charadriiformes identified to species were the European herring gull (39 vs 40), black-headed gull (37 vs 12), and mew gull (14 vs 5). The main Pelecaniformes identified to species were the grey heron (36 vs 58), great white egret (6 vs 1), and cattle egret (2 vs 1). The complete list of wild bird species found as HPAI-affected from 29 November 2025 to 27 February 2026 is reported in Figure A.1 in Annex A. The number of HPAI virus-affected wild birds that were not identified to species was 493/2108 (23%), more than 60% less than in the previous reporting period (2126/3370, 63%) (Figures A.1–A.2 in Annex A).

During the current reporting period, increased mortality of swans (likely mute swans) suspected or confirmed from HPAI continued to be observed at multiple locations in Croatia (Lake Jegeniš) (Veterina Portal, online), France (ici, online-a,b), Germany (LifePR, online; ProPlanta, online; Stern, online; Tag24, online), Hungary (Lake Balaton) (ZAOL, online), Moldova (IPN, online), Poland (Szczecin, online), Romania (Lake Prodana, Lake Bâtca Doamnei) (Europa FM, online; Flu Information Centre, online), Slovakia (Denník SME, online) and the United Kingdom (BBC, online; Express, online; Worcester News, online). Most reports were of mortality events of around 10 swans per location, but in the Camargue region of France around 50 swans were reported dead from HPAI. Apart from swans, mortality of cormorants was reported on the Baltic Sea in Germany (ProPlanta, online) and Poland (Szczecin, online). In Spain mortality of white storks from HPAI was reported, involving about 400 individuals in Madrid (SIC Noticias, online) and 32 breeding pairs at a breeding colony site of 96 pairs from Getafe (El País, online-a; TeleMadrid, online).

Still, this picture of HPAI-associated mortality of wild birds is incomplete, as reporting counts of dead wild birds in association with HPAI is not part of the current avian influenza surveillance system in Europe. Available data largely rely on voluntary efforts in some countries and media reports. Additionally, only a small proportion of wild birds found dead are submitted for HPAI testing. Therefore, HPAI virus detections in wild birds generally underestimate the number of wild birds actually dying from A(H5) virus infection.

In addition to the detections in dead birds, HPAI A(H5) virus continued to be detected in apparently healthy dabbling ducks (e.g. mallards, Eurasian wigeons and common teals in the Netherlands) in the current reporting period via avian influenza surveillance in live birds. Although the prevalence was lower (about 2–15% positives per week) than in the previous reporting period (up to about 30% positives per week), it still was higher than seen in previous years. In addition, HPAI A(H5) virus was also detected in apparently healthy birds of other species, including Caspian gulls and white-fronted geese (SENTINEL Wild Birds, online; personal communication by Ron Fourchier, Erasmus MC). This underlines the fact that HPAI surveillance in birds found dead only provides part of the picture of the epidemiology of HPAI in wildlife.

No new HPAI A(H5N5) virus detections were reported during the current reporting period. However, in addition to the data available via ADIS, the Danish national reference laboratory reported the detection of the HPAI A(H5N5) genotype EA-2021-I in a black-headed gull found dead in January in Denmark. HPAI A(H5N2) virus was identified in a single barnacle goose from Sweden. The same subtype was recently found in a hunted mallard from Latvia that had been sampled back in October 2025 via active surveillance (SENTINEL Wild Birds, online).

Note that Figures A.1–A.2 in Annex A provide information on the numbers of wild bird categories/families/species that were detected as HPAI-affected at single bird level, as more than one bird can be involved in a single HPAI virus detection reported.

#### 2.1.2 HPAI virus detections in birds outside Europe

An overview of HPAI virus detections in birds from countries outside Europe that were notified to WOAH from 29 November 2025 to 27 February 2026 is presented in Table 2 and Figure 6.

Table 2: Number of HPAI virus detections in non-European countries notified to WOAH, by virus subtype and country, from 29 November 2025 to 27 February 2026. Cumulative numbers since the start of the 2025–2026 epidemiological year are reported in parentheses (1 October to 27 February 2026).

| Region (total in season) | Country | Domestic birds | | | | Wild birds | | | | | Total |
|---|---|---|---|---|---|---|---|---|---|---|---|
| | | **A(H5N1)** | **A(H5N9)** | **A(H5Nx)** | **A (not typed)** | **A(H5N1)** | **A(H5N6)** | **A(H5N9)** | **A(H5Nx)** | **A (not typed)** | |
| Africa (34) | Namibia | - | - | - | - | 1 (1) | - | - | - | - | 1 (1) |
| | Nigeria | 6 (21) | - | - | - | - | - | - | - | - | 6 (21) |
| | South Africa | 2 (5) | - | - | - | 0 (7) | - | - | - | - | 2 (12) |
| Americas (435) | Argentina | 0 (1) | - | - | 1 (1) | - | - | - | - | 1 (1) | 2 (3) |
| | Brazil | 2 (2) | - | - | - | - | - | - | - | - | 2 (2) |
| | Canada | 21 (95) | - | - | - | - | - | - | - | - | 21 (95) |
| | Cayman Islands | 0 (1) | - | - | - | - | - | - | - | - | 0 (1) |
| | Colombia | 5 (7) | - | - | - | - | - | - | - | - | 5 (7) |
| | Guatemala | 0 (1) | - | - | - | - | - | - | - | - | 0 (1) |
| | Mexico | - | - | - | - | 0 (3) | - | - | - | - | 0 (3) |
| | United States of America | 145 (312) | - | 2 (5) | - | 1 (2) | - | - | 0 (1) | - | 148 (320) |
| | Uruguay | - | - | - | - | - | - | - | 1 (1) | 2 (2) | 3 (3) |
| Antarctica (1) | Heard Island and McDonald Islands | - | - | - | - | 1 (1) | - | - | - | - | 1 (1) |
| Asia (231) | Bhutan | 3 (3) | - | - | - | - | - | - | - | - | 3 (3) |
| | Cambodia | 0 (2) | - | - | - | - | - | - | - | - | 0 (2) |
| | China | - | - | - | - | 0 (1) | - | - | 0 (1) | - | 0 (2) |
| | India | 14 (14) | - | - | - | 5 (5) | - | - | - | - | 19 (19) |
| | Iraq | 1 (4) | - | - | - | - | - | - | - | - | 1 (4) |
| | Israel | 2 (2) | - | - | - | 5 (9) | - | - | - | - | 7 (11) |
| | Japan | 14 (18) | - | - | 0 (1) | 26 (73) | - | - | - | - | 40 (92) |
| | Mongolia | - | - | - | - | 0 (1) | - | - | - | - | 0 (1) |
| | Philippines | 1 (7) | - | 0 (1) | - | - | - | - | - | - | 1 (8) |
| | South Korea | 29 (34) | 3 (3) | - | - | 24 (34) | 0 (1) | 0 (2) | - | - | 56 (74) |
| | Taiwan | 12 (13) | - | - | - | 2 (2) | - | - | - | - | 14 (15) |
| Total | | **257 (542)** | **3 (3)** | **2 (6)** | **1 (2)** | **65 (139)** | **0 (1)** | **0 (2)** | **1 (3)** | **3 (3)** | **332 (701)** |

–, no HPAI outbreaks were notified via WOAH-WAHIS.

*Source*: WOAH-WAHIS (data extraction carried out on 27 February 2026).

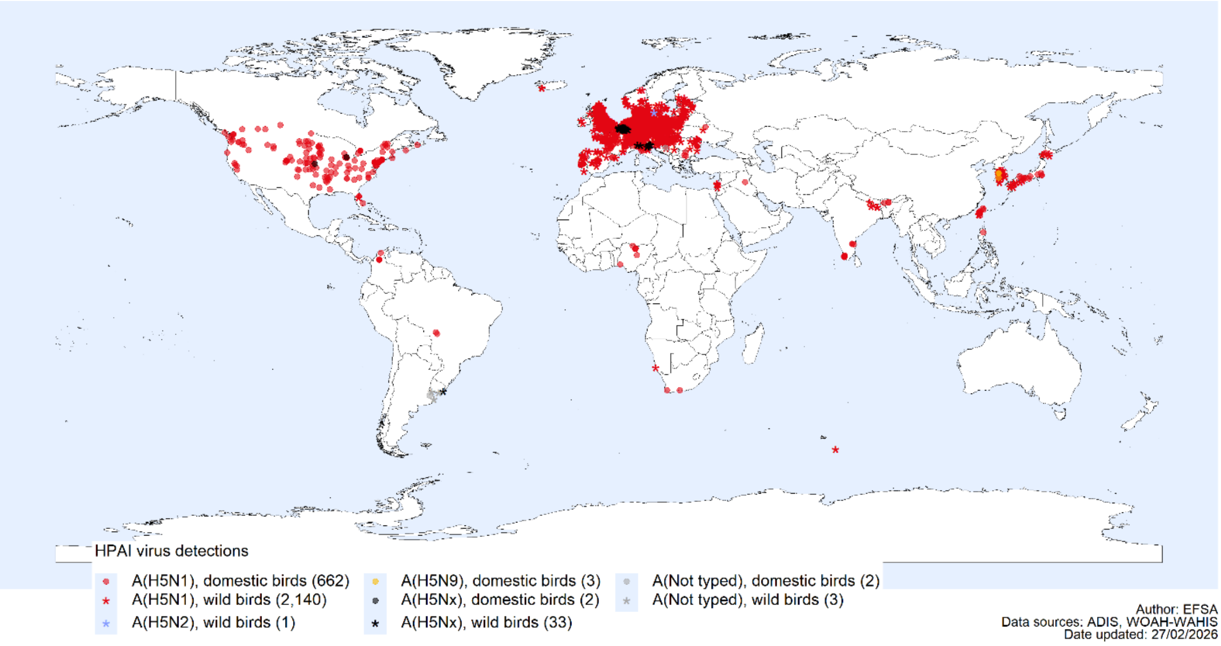

Figure 6: Geographic distribution, based on available geocoordinates, of HPAI virus detections reported worldwide in domestic (263) and wild (69) birds by virus type, from 29 November 2025 to 27 February 2026.

*Source*: ADIS and WOAH-WAHIS (data extraction carried out on 27 February 2026).

The tables and figures of the present report only include data extracted from WOAH-WAHIS on 27 February 2026. However, HPAI virus detections in domestic and wild birds are also reported to the public via different means. This additional information on HPAI virus detections available from sources other than WOAH-WAHIS has been integrated in the text below.

In comparison to the previous reporting period, the total number of HPAI virus detections in domestic and wild birds officially notified to WOAH from outside Europe decreased from 428 to 332, and also the number of reporting countries outside Europe decreased from 20 to 18 (Table 2, Figure 6). Compared to the same period in the previous epidemiological year, the number of official notifications to WOAH during the current reporting period was about 118% lower than the number of outbreaks reported between 29 November 2024 and 27 February 2025 (332 vs 724 outbreaks).

In Africa, the number of HPAI A(H5N1) outbreaks has decreased compared to the previous reporting period with 9 and 32 outbreaks, respectively. As in the previous reporting period (Table A.4 in Annex A), HPAI A(H5N1) outbreaks in domestic birds were reported to WOAH from South Africa. The epidemic in poultry continued, with this time small establishments affected (2,800 and 75 birds in the two outbreaks, respectively). In the current reporting period, HPAI A(H5N1) outbreaks in six poultry establishments (100 to 6,500 birds) were reported to WOAH also from West Africa (Nigeria). Between 29 November 2025 and 27 February 2026, only Namibia reported a wild bird outbreak in common terns.

Compared to the previous reporting period (Table A.4 in Annex A), the total number of HPAI virus detections in domestic and wild birds officially notified to WOAH from the United States of America (USA) and Canada decreased from 209 to 148 for the USA and from 81 to 21 for Canada. As in the previous reporting period, Canada only officially reported to WOAH HPAI virus detections in domestic birds. Among those were five outbreaks in captive birds (flocks of 6 to 600 birds) and 15 outbreaks in poultry, in both backyard and larger establishments (11 to close to 160,000 birds). The USA officially reported 64 outbreaks in captive birds (flocks of 7 to 605 birds), 144 outbreaks in poultry (wide range of establishment sizes) and one HPAI A(H5N1) outbreak in a grey parrot. The United States Department of Agriculture (USDA) reported 186 outbreaks in all types of poultry establishments between 29 November 2025 and 20 February 2026, mostly in turkeys and ducks for fattening on their website (USDA, online-a), including also 76 smaller or non-commercial backyard establishments. Furthermore, the USDA reported 1,584 HPAI virus detections in wild birds between 29 November 2025 and 20 February 2026 (USDA, online-b), substantially more than the detections officially notified to WOAH. Close to 90 wild bird species were affected, most of which were mallards (23.4%, 371/1584), Canada geese (20.6%, 327/1584) and snow geese (10%, 167/1584) (USDA, online-b). Indeed, in New York State, the media reported dead snow geese, Canada geese and ducks likely due to HPAI A(H5N1) (NBC New York, online). Pennsylvania, southwest Iowa, Delaware, and New Jersey also encountered HPAI A(H5N1) outbreaks according to the media with hundreds of dead snow geese (Independent, online), dozens of Canada geese (Des Moines Register, online), dozens of dead snow geese among other birds (Delaware Online, online), and > 1,100 dead or sick wild birds (mostly Canada geese) in the four states, respectively (Fox News, online). In Florida, the media reported on a dozen and then 36 dead swans at lake Eola in Orlando and on dead ducks in Torcaso Park likely because of HPAI A(H5N1) (Fox35, online-a,b; Outbreak News Today, online-a). During the current reporting period, no new HPAI A(H5N5) virus detections were reported in the USA or elsewhere outside Europe. In Canada, the media reported HPAI A(H5N1) in hundreds of Canada geese in Ontario (CBC, online-a, Chatham Daily News, online-a,b) and Manitoba (CBC, online-b; CityNews, online), in two dead whooping cranes in Saskatchewan (GlobalNews, online), and in dead crows (Chatham Daily News, online-c). Brazil and Colombia officially reported to WOAH two and five HPAI A(H5N1) outbreaks in captive birds, respectively, while Argentina officially notified HPAI virus detections in commercial breeders in Buenos Aires Province and in wild birds (black-necked swan, brown-hooded gull, Coscoroba swan, fulvous whistling duck) in an important wetland nearby (El País, online-b). Furthermore, three HPAI virus detections in Coscoroba swans were reported from Uruguay.

Between 29 November 2025 and 27 February 2026, Heard Island and McDonald Islands in Antarctica officially reported to WOAH an HPAI A(H5N1) outbreak in gentoo penguins. The media reported suspicions of HPAI A(H5N1) in gentoo penguins at O’Higgins Base but also in seabirds and penguins at Escudero Base (BiobioChile, online).

Outbreaks of HPAI A(H5) continued to be officially reported to WOAH from Asia and, in comparison to the previous reporting period, more countries were additionally affected by HPAI (H5), namely Bhutan and India (Table 2, Table A.4 in Annex A). In Bhutan, three small-sized poultry establishments (< 1000 birds) were affected. India officially notified WOAH of 14 HPAI A(H5N1) outbreaks in poultry (farms with 590 to 17,000 birds) and five in wild birds (five house crows). In addition, the media reported many cases of dead crows in different parts of the country: in Coimbatore (The Times of India, online-a), Tamil Nadu (The Health Site, online), Kannur (News18, online-a), and Bihar (News18, online-b). In the current reporting period, Iraq reported another HPAI A(H5N1) outbreak in an establishment keeping 60,000 birds, while Israel officially notified the detection of HPAI A(H5N1) in two poultry establishments as well as in five wild bird species (black-headed gull, common kestrel, great cormorant, yellow-legged gull). The media detailed that the outbreak occurred in a laying hen establishment in Baghdad Governorate and that contact with wild birds was identified as the most likely source of infection (Beacon, online-a). The media mentioned the infection of an 8000-turkeys establishment in the north of the country after an outbreak in a duck breeding site nearby early January (Archyde, online). In Japan, 14 and 26 HPAI A(H5N1) outbreaks in domestic and wild birds were reported to WOAH, respectively. The media detailed two of the domestic birds' outbreaks late January with large poultry establishments affected: a broiler establishment with more than 20,000 birds in Gifu Prefecture with 0.015% reported mortality, and a quail establishment with close to 110,000 birds in Chiba Prefecture with 0.23% reported mortality (Beacon, online-b). Most of the reports in wild birds corresponded to large-billed crows but also hooded cranes and different waterfowl species were affected. In the Philippines, one HPAI A(H5N1) outbreak was reported to WOAH in poultry. At the same time, South Korea officially notified to WOAH 32 and 24 HPAI A(H5) outbreaks in domestic and wilds birds, respectively. For poultry, affected establishments were small to very large with 8000 to 550,000 birds. For wild birds, waterfowl but also raptors were positive with the following species reported: peregrine falcon, grey heron, whooper swan, Eurasian eagle-owl, cinereous vulture, red-crested pochard, gadwall, greater white-fronted goose, great egret, Indian spot-billed duck, and mallard. Among the outbreaks in domestic birds, three were due to HPAI A(H5N9) viruses. In Taiwan, 12 outbreaks occurred in poultry (flocks of 5,000 to 60,000 birds and one backyard establishment) and two outbreaks were officially notified in wild birds (black-faced spoonbill, Eurasian wigeon). The media reported an outbreak in a large laying hen establishment in Taichung with 7000 birds culled (Beacon, online-c). The media also reported illegal disposal of more than 300 dead by dumping the carcasses into a fishpond in Yunlin County, after an HPAI A(H5N1) outbreak in commercial geese, raising biosecurity concerns geese (Beacon, online-d). Finally, the media reported HPAI A(H5N1)-infected backyard poultry early February in hundreds of domestic birds in Ha Tinh Province in Viet Nam (SGGP, online).

A list of all wild bird species that were reported to WOAH as HPAI-affected from outside Europe between 29 November 2025 and 27 February 2026 is presented in Table A.1 in Annex A.

#### 2.1.3 Genetic characteristics of HPAI viruses of the A(H5Nx) subtype in avian species

Details on the nomenclature of the HPAI A(H5) viruses used in this section are reported in Appendix B. Genotypes are assigned using the criteria described in Fusaro et al. (2024) and the tool GenIn2 (https://github.com/izsvenezie-virology/genin2). Mutation analyses of the HPAI A(H5N1) viruses have been performed using FluMut (Giussani et al., 2025) with the FluMutDB v6.4 mutation database (https://github.com/izsvenezie-virology/FluMutDB).

**Genetic diversity of HPAI A(H5Nx) viruses in avian species in Europe**

Since October 2025, more than 1350 complete genome sequences of European viruses from 25 countries have been characterised. All the viruses belong to clade 2.3.4.4b, which is the only clade detected in Europe since 2016.

Based on the genetic data available as of 3 March 2026, during the 2025–2026 epidemiological year, the vast majority (> 90%) of the sequenced viruses belonged to a single genotype, EA-2024-DI, subgenotype EA-2024-DI.2.1. This subgenotype has been detected in wild (mainly Anseriformes) and domestic birds from all the 25 European countries for which data are available, representing the most widespread genotype in Europe, extending north from Iceland to Finland and south from Portugal to Bulgaria, reaching as far as the Republic of Georgia, where it has been associated with mass mortality events in great crested grebes along the Black Sea coast. Outside Europe, the EA-2024-DI.2.1 genotype has been identified in October 2025 in wild Anseriformes in Kazakhstan.

Genetic data indicate that this genotype was also responsible for the mass mortality events reported in common cranes from some of the countries along its main European migratory route from Sweden to Spain. Of note, the vast majority of the characterised viruses from common cranes collected from Sweden, Poland, Germany, Belgium, Netherlands, Luxembourg, France and Spain cluster together, suggesting a single virus introduction in this species followed by intra-species transmission. This cluster includes also viruses collected in domestic birds as well as in other wild bird and mammalian species, suggesting virus spread to other hosts directly from common cranes or through a highly contaminated environment.

Beside EA-2024-DI.2.1, since October 2025, A(H5N1) genotypes circulating in the previous epidemic waves, namely EA-2024-DI.2, EA-2024-DT, EA-2022-BB, EA-2023-DA and EA-2021-I, have been occasionally identified in few geographical areas. Genotype EA-2024-DI.2 has been detected in wild (mainly Anseriformes) and domestic birds in Western Europe: the United Kingdom, Spain and Portugal. However, viruses collected in the United Kingdom cluster separately from those identified in Spain and Portugal, suggesting a persistent but separate circulation of this genotype in these two geographical areas. Genotypes EA-2024-DT and EA-2022-BB continue to circulate among Laridae and other see birds (ie. sanderlings) in separate geographical areas: EA-2024-DT in Spain and Portugal; EA-2022-BB in Belgium, Norway and the United Kingdom. Genotype EA-2023-DA continues to be detected exclusively in poultry in Bulgaria and Czechia; this genotype has not been detected in wild birds during the previous (2024–2025) and the current epidemiological year in or outside other regions of Europe. The viruses collected in Bulgaria and Czechia cluster separately in the phylogenetic trees suggesting an ongoing separate circulation in an unknown ecological or domestic niche in the two countries. The A(H5N5) genotype EA-2021-I was detected at the end of January in a black-headed gull in Denmark, after about 3 months from its last probable identification in a northern fulmar in the Netherlands (only partial genome sequences available) and 6 months from its identification in wild birds and mammals in Norway. This represents the first identification of genotype EA-2021-I in this country. This virus falls within a group of A(H5N5) viruses that have been circulating in Northern Europe since November 2024, all characterised by the PB2-E627K molecular marker. Notably, the recent identification (January 2026) of a virus belonging to this European cluster in a carrion crow in Japan suggests a novel virus spread of this strain from Europe to Japan.

In addition to the known genotypes, several reassortant viruses, in almost all cases originating from reassortment events involving mainly the PB2, PB1 or NP gene segments between the EA-2024-DI.2.1 and LPAI viruses, have been characterised in Europe, including an A(H5N2) from a barnacle goose from Sweden. All these new reassortants have been identified in one or very few outbreaks within one or two countries, indicating their scarce epidemiological relevance.

**Mutations identified in HPAI A(H5Nx) viruses from avian species in Europe**

Molecular analyses of the HPAI A(H5N1) viruses circulating in birds in Europe since October 2024 indicate that these viruses do not contain critical mutations detected in previous pandemic strains in the receptor binding site of the HA protein, meaning that they continue to be well-adapted to avian species. However, a recent study (Weber et al., 2026) showed that clade 2.3.4.4b viruses have the unique ability to bind mucin-like O-glycans, which are not recognised by other A(H5) clades. Although these viruses maintain an avian-type receptor specificity, their wide host range can potentially be explained by their broad receptor specificity.

Moreover, several mutations previously described in literature (Du et al., 2018, 2021; Suttie et al., 2019; Pinto et al., 2023) have been identified as being associated with i) enhanced polymerase activity and replication in mammals or mammalian cells, ii) increased virulence, iii) increased/conferred resistance towards antiviral drugs, iv) increased in vitro binding to human-type receptors α2,6-SA, v) decreased antiviral response in ferrets, vi) evasion of human butyrophilin subfamily 3 member A3 (BTN3A3), and vii) disruption of the second sialic acid (SIA) binding site in the neuraminidase protein, with a frequency varying by distinct mutations. The net effect of these mutations on the biological characteristics of the viruses is still unknown, and further research is needed to improve existing knowledge.

Similarly to the viruses collected during the previous epidemiological years (2020–2021, 2021–2022, 2022–2023, 2023-2024, 2024–2025), the circulating European viruses contain some of the mutations in the receptor binding domain of the HA protein that have been shown by in vitro assays studies to promote, to a certain extent, the recognition of human-type receptors of specific H5 viruses. Specifically, some of them (i.e. S133A, S154N, T156A and H5 numbering) have been identified in the majority of the 2025–2026 A(H5N1) viruses, whereas others (i.e. D94N, G139R, HA1:S123P and HA2:R167K T188I, N189D, P235S, E251K and S155N) have only been sporadically observed (≤ 40 viruses). The impact of all these HA mutations on the biological characteristics of the circulating viruses is still unknown. Besides the mutations in the HA protein, it is important to mention that most of the A(H5N1) viruses belonging to the EA-2022-BB and EA-2023-DT genotypes contain mutations NP-Y52N and NA-S369I, which may increase their zoonotic potential due to the evasion of innate immunity and mammalian restriction factors or disruption of the second sialic acid binding site (2SBS). All the currently circulating A(H5N5) viruses (genotype EA-2021-I) contain a deletion in the NA stalk region, which is a virulence determinant in chickens (Stech et al., 2015). Additionally, all the A(H5N1) viruses of genotype EA-2024-DI.2.1 detected in Europe since mid-September 2025 contain a truncated NS1, 217 amino acids long, resembling that of human influenza A(H1N1) (219 amino acid in length). Such truncation of 13 amino acids has been observed in only 3.5% of the European A(H5N1) viruses of the 2024–2025 epidemiological year. An in vivo and in vitro study (Blaurock et al., 2021) showed that A(H5N8) viruses of clade 2.3.4.4b with a shorter NS1 are more efficient at blocking apoptosis and IFN-β response without a significant impact on virus replication in human cells and are capable to spread more efficiently among human and avian cells. The role of the variation in the NS1 lengths in the virulence of avian influenza viruses in birds and mammals is controversial and highly strain-dependent.

Mutations associated with a reduced susceptibility of A(H5N1) viruses to the available antiviral drugs authorised for use in humans have rarely been identified in the circulating strains. Specifically, mutations associated with resistance to amantadine and rimantadine, NA and PA inhibitors (WHO, online-a) have been detected in approximately 1.2%, 0.5% and 0.1% of the analysed A(H5Nx) viruses collected in Europe since October 2025 (n > 1350), respectively. However, for PA inhibitors, it is important to mention that mutations have been studied in seasonal influenza viruses and not specifically in A(H5N1) viruses; the threshold of ≥ 3 $IC_{50}$ fold-change was used for the assignment of viruses with a reduced susceptibility to PA inhibitors.

Since October 2025, the K526R, E627K and D701N mutations in the PB2 protein associated with virus adaptation in mammals (Suttie et al., 2019) have been detected in seven European outbreaks in birds, more specifically in i) 6 A(H5N1) viruses collected from domestic and captive birds from 5 different countries and ii) 1 A(H5N5) virus (genotype EA-2021-I) collected from a wild bird in Denmark. Based on the available data, a decreased frequency of detection of such mutations has been observed in birds, from about 4% in the 2024–2025 epidemiological year to about 0.4% in the 2025–2026 epidemiological year (based on data available up to 3 March 2026). Of note, all the A(H5N5) viruses of genotype EA-2021-I that have been collected in Europe since November 2024 contain the PB2 E627K/V molecular marker of mammalian adaptation, indicating that such mutation become fixed in this virus population.

### 2.2 HPAI virus detections in non-human mammals

Between 29 November 2025 and 13 February 2026, HPAI A(H5N1) viruses were reported in wild, pet and domestic mammals both in and outside Europe. Corresponding to the observed high circulation of HPAI viruses in wild birds during the current epidemiological year, the number of detections in mammals slightly increased. At the same time, no new HPAI A(H5N5) virus detections were reported during the current reporting period. The data described in this report were actively collected from Member States and other European countries, retrieved via WOAH-WAHIS and from the USDA websites (for cases that occurred in the USA), and supplemented with information from media reports (Table 3).

In Europe, HPAI A(H5N1) virus was predominantly reported in wildlife during the current reporting period. A total of 11 dead red foxes were found positive via passive surveillance in Germany (10) and the Netherlands (1), while in Estonia (1) and France (1) (ici, online-c), 2 red foxes exhibiting neurological signs tested positive. Denmark (1) and Spain (1) retrospectively reported HPAI A(H5N1) virus in 2 red foxes found dead via passive surveillance that were sampled during the previous reporting period. Germany also reported positive HPAI A(H5N1) virus detections in 10 dead raccoons found via passive surveillance between 29 November 2025 and 13 February 2026. In addition, a juvenile harbour seal that was found dead on a beach in the Netherlands tested positive for the same virus (RTV NOF, online). In Sweden, a captive harbour seal at a zoo tested positive to HPAI A(H5N1) virus after developing clinical signs (lethargy, decrease in feed intake, and strabismus) and dying. No other mammals or birds at the zoo were affected, and the most likely source of infection for this animal was contact with wild birds.

As regards pets and domestic mammals, for the first time in Europe, the first potential spillover of HPAI A(H5N1) virus from wild birds into dairy cattle was suggested by serology in the Netherlands (Wageningen University & Research, online). The investigation was triggered following the detection of HPAI A(H5N1) virus in a sick farm cat on 24 December 2025. Twenty individual dairy cattle and the bulk tank milk on the affected farm were sampled on 15 January 2026. At the time of sampling, the herd appeared clinically healthy. Molecular testing (PCR) detected no viral genome, but serological testing revealed the presence of A(H5N1) virus-specific antibodies. After this finding the entire herd was sampled; individual milk samples were collected from all lactating cows, and blood samples were collected from these animals and non-lactating youngstock present on the same farm. The serological results indicated a previous infection that had since faded out. The cattle in this farm had remained on pasture until late November 2025 (late in the season for Dutch grazing standards). The farm is located in a region with high waterfowl density and mortality among these birds was reported by the farmer. The closest confirmed HPAI A(H5N1) virus detection was approximately 2 km away from the impacted farm. In response to these findings, a retrospective study is underway. Historical serum samples from neighbouring farms and a nationwide representative set are currently being screened to determine the extent of the spillover. Results from this survey are pending.

At the same time, several European countries with variable testing efforts actively reported the absence of HPAI viruses in mammals during the current reporting period: Finland, Luxembourg,

Moldova, Norway, and Switzerland. Austria moreover shared detailed test results of analyses performed in mammals, all of which resulted negative: wild boar (162), bat (70), pig (56), fox (10), badger (2), and cat (2) (personal communication by Sandra Revilla-Fernández, AGES).

Similarly, the majority of HPAI A(H5N1) virus detections in mammals outside Europe can be attributed to wildlife. In the USA, according to the USDA, a range of different species were affected between 29 November 2025 and 13 February 2026 (USDA, online-c): Norway rats (brown rats) (5), Catalina island foxes (a subspecies of the island fox) (4), raccoons (4), mountain lions (3), red foxes (3), bobcats (2), bottlenose dolphins (2), skunks (2), striped skunks (2), fox (1) and house mouse (1). The media mentioned mortality of 29 northern elephant seal pups and 1 adult individual in California, where animals exhibited neurological and respiratory signs. HPAI A(H5N1) virus was detected in 7 of the dead pups (Los Angeles Times, online). Ongoing mortality of Antarctic fur seals in Antarctica has been reported, with no proven involvement of HPAI (BiobioChile, online).

As regards pets, 4 more HPAI A(H5N1) virus detections in domestic cats were reported in the USA during the current reporting period (USDA, online-c), while the number of dairy cattle farms reportedly affected by the epidemic rose by 5 to 1,088 in 19 states (for the first time in Wisconsin) (USDA, online-d). Recent detections in dairy cattle in the USA now also involve the D1.1 genotype, in addition to the predominant B3.13 genotype, demonstrating that additional spillover events have occurred (Kelo, online). At least three isolated spillover events of the D1.1 genotype have been detected in Nevada, Arizona and Wisconsin since the beginning of the year. In Canada, at the end of 2025, a domestic dog that had reportedly fed on a snow goose was found positive to HPAI A(H5N1) virus after showing neurological and respiratory signs (BNO News, online).

Table 3: Avian influenza A(H5Nx) virus detections in mammalian species other than humans related to circulating viruses worldwide, 2016–2026.

| Virus | Animal (order, family, species) | | | Country | Source |
|---|---|---|---|---|---|
| A(H5N1) or A(H5Nx) clade 2.3.4.4b | Artiodactyla | Bovidae | Cattle (*Bos taurus*) | Netherlands[a], United States of America | WOAH<br>USDA<br>Wageningen University and Research (online) |
| | | | Goat (*Capra hircus*) | United States of America | WOAH<br>USDA |
| | | | Sheep (*Ovis aries*) | Norway, United Kingdom | Fosse et al. (2025), GovUK (online-a,b) |
| | | Camelidae | Alpaca (*Lama pacos*) | United States of America | USDA (online-d) |
| | | Suidae | Pig (*Sus scrofa*) | Italy[a], United States of America | WOAH<br>Rosone et al. (2023) |
| | Carnivora | Canidae | Arctic fox (*Vulpes lagopus*) | Finland | WOAH |
| | | | Common raccoon dog (*Nyctereutes procyonoides*) | Finland, Japan, Sweden[a] | WOAH |
| | | | Coyote (*Canis latrans*) | United States of America | WOAH<br>USDA |
| | | | Dog (*Canis lupus familiaris*) | Canada, Italy[a], Poland | WOAH<br>Szaluś-Jordanow et al. (2024) |
| | | | Gray fox (*Urocyon cinereoargenteus*) | United States of America | USDA |
| | | | Island fox (*Urocyon littoralis*) | United States of America | USDA |
| | | | Japanese raccoon dog (*Nyctereutes viverrinus*) | Japan | WOAH |
| | | | Red fox (*Vulpes vulpes*) | Austria, Belgium, Canada, Denmark, | WOAH |

| Virus | Animal (order, family, species) | | | Country | Source |
|---|---|---|---|---|---|
| | | | | Estonia, Finland, France, Germany, Ireland, Italy, Japan, Latvia, Netherlands, Norway, Slovenia, Spain, Sweden[b], United Kingdom, United States of America | Personal communication by Aleksandra Hari (AFSVSPP)<br>Personal communication by Sandra Revilla-Fernández (AGES) |
| | | | South American bush dog (*Speothos venaticus venaticus*) | United Kingdom | WOAH |
| | | Felidae | Bobcat (*Lynx rufus*) | United States of America | WOAH<br>USDA |
| | | | Canadian lynx (*Lynx canadensis*) | United States of America | WOAH<br>USDA |
| | | | Caracal (*Caracal caracal*) | Poland, United States of America | WOAH<br>USDA |
| | | | Cat (*Felis catus*) | Canada, Belgium, France[b], Germany, Hungary[b], Italy[a], Netherlands[b], Poland, South Korea, United States of America | WOAH<br>USDA<br>DutchNews (online), TF1 Info (online)<br>Personal communication by Ingeborg Mertens (FAVV) |
| | | | Cheetah (*Acinonyx jubatus*) | United States of America | USDA |
| | | | Eurasian lynx (*Lynx lynx*) | Finland, Sweden[a], United States of America | WOAH<br>USDA |
| | | | Leopard (*Panthera pardus*) | India, United States of America, Viet Nam | USDA<br>The Times of India (online-b) |
| | | | Leopard cat (*Prionailurus bengalensis*) | South Korea | WOAH |
| | | | Lion (*Panthera leo*) | India, Peru, United States of America, Viet Nam | WOAH<br>USDA<br>Hindustan Times (online), The Times of India (online-c) |
| | | | Mountain lion (*Puma concolor*) | United States of America | WOAH<br>USDA |
| | | | Serval (*Leptailurus serval*) | Bangladesh, United States of America | WOAH<br>USDA |
| | | | Tiger (*Panthera tigris*) | India, United States of America, Viet Nam | WOAH<br>USDA<br>Hindustan Times (online), The Times of India (online-b,c) |
| | | Mephitidae | Striped skunk (*Mephitis mephitis*) | Canada, United States of America | WOAH<br>USDA |
| | | Mustelidae | American marten (*Martes americana*) | United States of America | WOAH<br>USDA |
| | | | American mink (*Neovison vison*) | Canada, Finland, Spain, United States of America | WOAH<br>USDA |
| | | | Beech marten (*Martes foina*) | Netherlands | GISAID (online) |
| | | | Eurasian otter (*Lutra lutra*) | Netherlands, Finland, France, Sweden, United Kingdom | WOAH<br>Préfet des Landes (online) |
| | | | European badger (*Meles meles*) | Netherlands | WOAH |

| Virus | Animal (order, family, species) | | | Country | Source |
|---|---|---|---|---|---|
| | | | European pine marten (*Martes martes*) | Germany | WOAH |
| | | | European polecat (*Mustela putorius*) | Belgium, Netherlands | WOAH |
| | | | Ferret (*Mustela furo*) | Belgium, Poland, Slovenia | WOAH<br>Golke et al. (2024) |
| | | | Fisher (*Pekania pennanti*) | United States of America | WOAH<br>USDA |
| | | | Long-tailed weasel (*Neogale frenata*) | United States of America | USDA |
| | | | Marine otter (*Lontra felina*) | Chile | WOAH |
| | | | North American river otter (*Lontra canadensis*) | United States of America | WOAH |
| | | | Southern river otter (*Lontra provocax*) | Chile | WOAH |
| | | | Stoat (*Mustela erminea*) | United States of America | WOAH<br>USDA |
| | | Odobenidae | Walrus (*Odobenus rosmarus*) | Norway | WOAH |
| | | Otariidae | Antarctic fur seal (*Arctocephalus gazella*) | South Georgia and the South Sandwich Islands, Uruguay | WOAH<br>Banyard et al. (2024), Bennison et al. (2024) |
| | | | Northern fur seal (*Callorhinus ursinus*) | Russia | WOAH |
| | | | South American fur seal (*Arctocephalus australis*) | Argentina, Brazil, Peru, Uruguay | WOAH |
| | | | South American sea lion (*Otaria flavescens*) | Argentina, Brazil, Chile, Peru, Uruguay | WOAH |
| | | Phocidae | Caspian seal (*Pusa caspica*) | Russia | WOAH |
| | | | Crabeater seal (*Lobodon carcinophaga*) | Joinville Island | Phys.org (online) |
| | | | Grey seal (*Halichoerus grypus*) | Canada, Germany, Netherlands, Poland, Sweden[b], United Kingdom, United States of America | WOAH |
| | | | Harbour seal (*Phoca vitulina*) | Canada, Denmark, Germany, Japan, Netherlands, South Georgia and the South Sandwich Islands, Sweden, United Kingdom, United States of America | WOAH<br>USDA<br>The Mainichi (online) |
| | | | Northern elephant seal (*Mirounga angustirostris*) | United States of America | Los Angeles Times (online) |
| | | | Southern elephant seal (*Mirounga leonina*) | Argentina, South Georgia and the South Sandwich Islands, Possession Island | WOAH<br>Banyard et al. (2024), Bennison et al. (2024), PASTAAF (online-a,b) |
| | | Procyonidae | Raccoon (*Procyon lotor*) | Canada, Germany, United States of America | WOAH<br>USDA |
| | | | South American coati (*Nasua nasua*) | Germany, Uruguay | WOAH |
| | | Ursidae | American black bear (*Ursus americanus*) | Canada, United States of America | WOAH<br>USDA |
| | | | Asian black bear (*Ursus thibetanus*) | France | WOAH |
| | | | Brown bear (*Ursus arctos*) | United States of America | WOAH |
| | | | Kodiak grizzly bear (*Ursus arctos horribilis*) | United States of America | WOAH |

| Virus | Animal (order, family, species) | | | Country | Source |
|---|---|---|---|---|---|
| | | | Polar bear (*Ursus maritimus*) | United States of America | WOAH<br>USDA |
| | Cetacea | Delphinidae | Bottlenose dolphin (*Tursiops truncatus*) | Peru, United States of America | WOAH<br>USDA |
| | | | Chilean dolphin (*Cephalorhynchus eutropia*) | Chile | WOAH |
| | | | Common dolphin (*Delphinus delphis*) | Peru, United Kingdom | WOAH<br>Leguia et al. (2023) |
| | | | White-sided dolphin (*Lagenorhynchus acutus*) | Canada | WOAH |
| | | Phocoenidae | Burmeister's porpoise (*Phocoena spinipinnis*) | Chile | WOAH |
| | | | Harbour porpoise (*Phocoena phocoena*) | Sweden, United Kingdom | WOAH |
| | Didelphimorphia | Didelphidae | Virginia opossum (*Didelphis virginiana*) | United States of America | WOAH<br>USDA |
| | Lagomorpha | Leporidae | Desert cottontail (*Sylvilagus audubonii*) | United States of America | USDA |
| | Rodentia | Cricetidae | Deer mouse (*Peromyscus* spp.) | United States of America | USDA |
| | | | Muskrat (*Ondatra zibethicus*) | United States of America | USDA |
| | | | Prairie vole (*Microtus ochrogaster*) | United States of America | USDA |
| | | Muridae | Black rat (*Rattus rattus*) | United States of America | USDA |
| | | | Brown rat (*Rattus norvegicus*) | Egypt, United States of America | USDA<br>Kutkat et al. (2024) |
| | | | House mouse (*Mus musculus*) | United States of America | WOAH<br>USDA |
| | | | House rat (*Rattus rattus*) | Egypt | Kutkat et al. (2024) |
| | | Sciuridae | Abert's squirrel (*Sciurus aberti*) | United States of America | WOAH |
| | | | Eastern gray squirrel (*Sciurus carolinensis*) | United States of America | WOAH<br>USDA |
| | | | Round-tailed ground squirrel (*Xerospermophilus tereticaudus*) | United States of America | USDA |
| | | | Thirteen-lined ground squirrel (*Ictidomys tridecemlineatus*) | United States of America | USDA |
| **A(H5N1) clade 2.3.2.1a** | Carnivora | Felidae | Cat (*Felis catus*) | India | Raut et al. (2025)<br>The Times of India (online-d) |
| **A(H5N5) clade 2.3.4.4b** | Carnivora | Canidae | Arctic fox (*Vulpes lagopus*) | Iceland, Norway | WOAH<br>Personal communication by Brigitte Brugger (MAST, 2025)<br>Personal communication by Ingeborg Slettebø Wathne (Norwegian Food Safety Authority, 2025) |

| Virus | Animal (order, family, species) | | | Country | Source |
|---|---|---|---|---|---|
| | | | Red fox (*Vulpes vulpes*) | Canada, Norway | WOAH |
| | | Felidae | Bobcat (*Lynx rufus*) | Canada | WOAH |
| | | | Cat (*Felis catus*) | Canada, Iceland | WOAH<br>Personal communication by Brigitte Brugger (MAST, 2025) |
| | | | Eurasian lynx (*Lynx lynx*) | Norway | Personal communication by Silje Granstad (Norwegian Veterinary Institute, 2025) and Lars-Erik Lund Rondestveit (Norwegian Food Safety Authority, 2025) |
| | | Mephitidae | Striped skunk (*Mephitis mephitis*) | Canada | WOAH<br>CFIA (online) |
| | | Mustelidae | American mink (*Neovison vision*) | Iceland | Personal communication by Brigitte Brugger (MAST, 2025) |
| | | | Eurasian otter (*Lutra lutra*) | Norway | Personal communication by Silje Granstad (Norwegian Veterinary Institute, 2025) and Lars-Erik Lund Rondestveit (Norwegian Food Safety Authority, 2025) |
| | | | European pine marten (*Martes martes*) | Netherlands | Personal communication by Dennis Bol (NVWA, 2025) |
| | | Phocidae | Grey seal (*Halichoerus grypus*) | United Kingdom | WOAH<br>FarmingUK (online), GovUK (online-c) |
| | | | Ringed seal (*Pusa hispida*) | Canada | WOAH<br>CFIA (online) |
| | | Procyonidae | Raccoon (*Procyon lotor*) | Canada | WOAH |
| **A(H5N6) clade 2.3.4.4b** | Carnivora | Canidae | Dog (*Canis lupus familiaris*) | China | Yao et al. (2023) |
| | | Mustelidae | American mink (*Neovison vison*) | China | Zhao et al. (2024) |
| **A(H5N8) clade 2.3.4.4b** | Artiodactyla | Suidae | Pig (domestic) (*Sus scrofa*)[a] | France | Herve et al. (2021) |
| | | | Pig (wild boar) (*Sus scrofa*)[a] | Germany | Schülein et al. (2021) |
| | Carnivora | Canidae | Red fox (*Vulpes vulpes*) | United Kingdom | WOAH |
| | | Phocidae | Grey seal (*Halichoerus grypus*) | Poland, Sweden, United Kingdom | SVA<br>Shin et al. (2019), Floyd et al. (2021)<br>Personal communication by Siamak Zohari (SVA, 2024) |
| | | | Harbour seal (*Phoca vitulina*) | Denmark, Germany, United Kingdom | WOAH<br>Floyd et al. (2021), Ärzteblatt (online), Avian Flu Diary (online), Outbreak |

| Virus | Animal (order, family, species) | | | Country | Source |
|---|---|---|---|---|---|
| | | | | | News Today (online-b) |

[a] Serological detection.
[b] Both virological and serological detection.

#### 2.2.1 Genetic characteristics of HPAI viruses of the A(H5Nx) subtype circulating in Europe in non-human mammals

Details on the nomenclature of the HPAI A(H5) viruses used in this section are reported in Appendix B. Genotypes are assigned using the criteria described in Fusaro et al. (2024) and the tool GenIn2 (https://github.com/izsvenezie-virology/genin2). Mutation analyses of the A(H5N1) viruses have been performed using FluMut (Giussani et al., 2025) with FluMutDB v6.4 mutation database (https://github.com/izsvenezie-virology/FluMutDB).

Based on the genetic data available as of 3 March 2026, since October 2025, the complete or partial genome sequences of 20 HPAI A(H5N1) viruses of clade 2.3.4.4b collected from four distinct mammalian species (i.e. domestic cat, Eurasian otter, harbour seal and red fox) in eight different European countries have been analysed, including the virus from a dead domestic cat collected from the serologically positive dairy cattle farm in the Netherlands. All the characterised viruses belong to the A(H5N1) genotype EA-2024-DI.2.1. Specifically, the A(H5N1) viruses collected from wild mammals (7 red foxes and 1 Eurasian otter) in France, Germany and Spain cluster together with viruses associated with the mass mortality events in common cranes in these countries.

Of note, 17/20 viruses (85%) contain the PB2 molecular marker of mammalian adaptation E627K, which have not been identified in the phylogenetically related viruses collected from avian species. Additionally, one virus acquired the PB2 substitutions E192K and Q591K (Yamada et al., 2010; Taft et al., 2015).

### 2.3 Avian influenza virus infections in humans

#### 2.3.1 Overview of the most recent human infections with avian influenza viruses

Since 29 November 2025 and as of 27 February 2026, 10 new human cases of infection with avian influenza viruses have been reported from two countries: Cambodia (n = 1) and China (n = 9). These infections were caused by three different subtypes: A(H5N1), A(H9N2) and A(H10N3) (Table 4). Most of the cases had exposure to poultry or live animal markets (Table 5).

Table 4: Globally reported cases or detections of avian influenza virus in humans, including virus subtypes reported in the last 12 months.

| Subtype | Cases reported 2025-11-29–2026-02-27 | | | Cases reported since first report | | | |
|---|---|---|---|---|---|---|---|
| | Cases reported | Deaths | Reporting countries | First report | Cases reported | Deaths | Reporting countries |
| A(H5N1) | 1 | 0 | 1 | 1997 | 1 014[a] | 476[b] | 25 |
| A(H5N2) | 0 | 0 | 0 | 2024 | 2 | 1 | 1 |
| A(H5N5) | 0 | 0 | 0 | 2025 | 1 | 1 | 1 |
| A(H9N2) | 8 | 0 | 1 | 1998 | 195 | 2 | 10 |
| A(H10N3) | 1 | 0 | 1 | 2021 | 7 | 0 | 1 |

[a] Human cases of A(H5) epidemiologically linked to A(H5N1) outbreaks in poultry and dairy cattle farms in the USA are included in the reported number of A(H5N1) cases.
[b] Deaths among a total of 994 cases reported 2003–27 February 2026. Mortality data are not available for cases reported prior to 2003.

*Source*: Line list maintained by ECDC.

Table 5: Identified exposure associated with human cases of avian influenza, reported globally 29 November 2025–27 February 2026 by zoonotic influenza subtype.

| | Reported exposure[a] | Cases |
|---|---|---|
| **A(H5N1)** | Poultry | 1 |
| **A(H9N2)** | Poultry | 5 |
| | Domestic parrot | 2 |
| | Not reported[b] | 3 |
| **A(H10N3)** | Poultry | 1 |

[a] Exposure to more than one animal species can be reported for cases.
[b] Not reported or not publicly available.

*Source*: Line list maintained by ECDC.

#### 2.3.2 Human A(H5N1) cases

Between 29 November 2025 and 27 February 2026, one new human case of influenza A(H5N1) virus infection was reported from Cambodia. The case was reported from the Kampot province in Cambodia (CHP, online-a; WHO, online-b,c). The case was in an adult in the age range 30–39 years without reported underlying conditions, who was discharged from hospital on 14 February 2026 following recovery. The reported symptoms included fever, cough and abdominal pain. The patient had had exposure to poultry, with deaths reported in the household's flock of backyard poultry. No additional cases have been reported among close contacts. The sequence of the virus was not publicly available.

Since the first report of human infection with avian influenza A(H5N1) virus in 1997, a total of 1014 cases have been reported to the World Health Organization (WHO) from 25 countries (Figure 7). Since 2003, there have been 476 deaths out of a total of 994 cases reported.

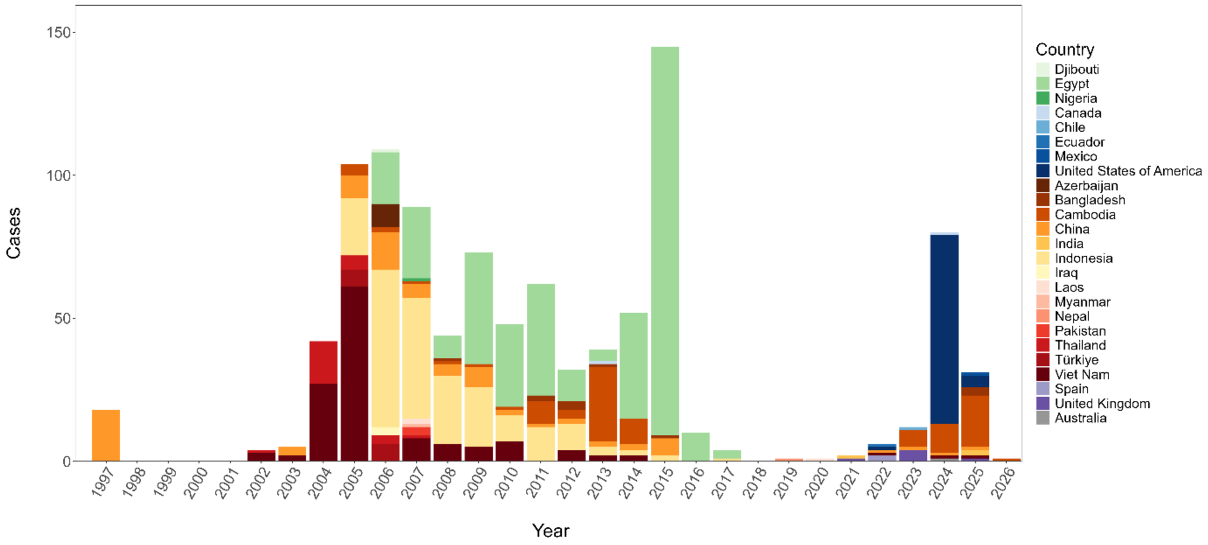

Figure 7: Distribution of reported human cases of A(H5N1) virus infection by year of onset or detection and reporting country from 1997 to 27 February 2026.

*Note*: The figure includes detections of A(H5N1) due to suspected environmental contamination reported in 2022 by Spain (2) and the USA (1), and in 2023 by the United Kingdom (three detections, one inconclusive). Human cases of A(H5) epidemiologically linked to A(H5N1) outbreaks in poultry and dairy cattle farms in the USA are included in the number of A(H5N1) cases.

*Source*: Line list maintained by ECDC.

#### 2.3.3 Human A(H9N2) cases

Between 29 November 2025 and 27 February 2026, eight new human cases of influenza A(H9N2) virus infection were reported from China (CHP, online-b,c,d; WHO, online-d,e,f). The cases were reported from the Guangxi Zhuang Autonomous Region and the Guangdong, Hubei, Hunan, and Jiangsu Provinces. The cases were in five children (age range 1−10 years) and three adults (over 70 years). The adults experienced a severe course of the disease, while the children had a mild disease. Most cases had direct or indirect exposure to poultry or fresh poultry meat. All cases recovered. Investigations of the cases did not reveal any epidemiological links between them. For some cases, the virus was detected in environmental samples collected during the investigations.

Since the first report of human infection with avian influenza A(H9N2) virus in 1998, a total of 195 cases, including two deaths, have been reported to WHO (Figure 8).

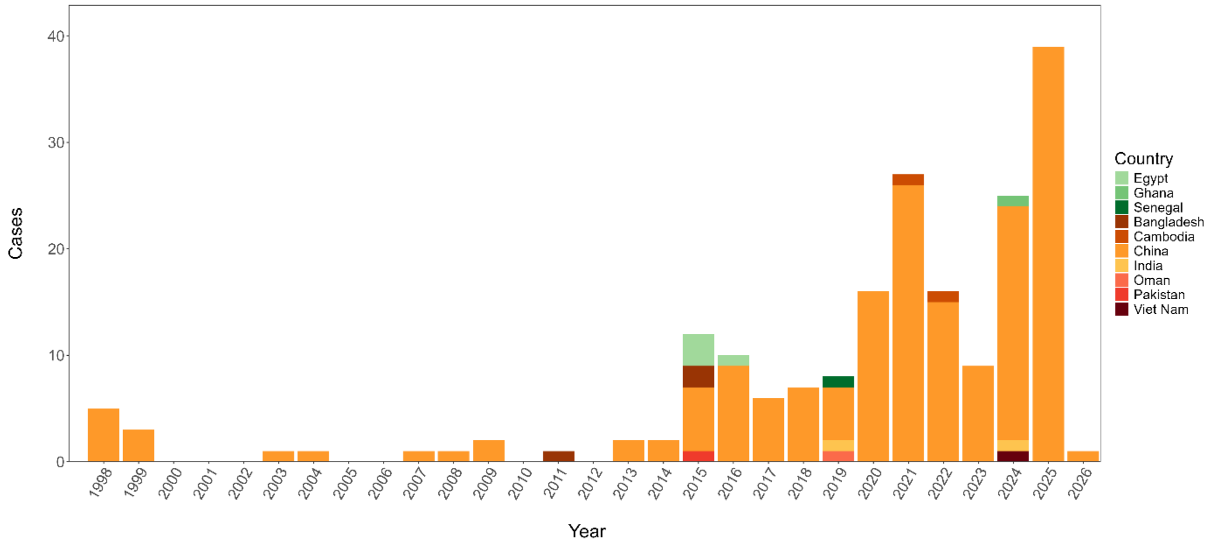

Figure 8: Distribution of reported human cases of A(H9N2) virus infection by year of onset or detection and reporting country, from 1998 to 27 February 2026.

*Source*: Line list maintained by ECDC.

The sequence of one of these A(H9N2) viruses, A/Suzhou/1209KS/2025, was publicly available. The sample was collected on 9 December 2025 in the Jiangsu Province. The sequence was retrieved and identified as clade B4.7.2. Most mutations detected in the strain have previously been observed in genetically related strains from human cases – e.g. A/Changsha/SR353/2025, described in a previous report (EFSA, ECDC and EURL, 2025a). This includes Q222L in the HA (H5 numbering), associated with enhanced replication and contact transmission in ferrets (Suttie et al., 2019). A/Suzhou/1209KS/2025 had not acquired V or K substitution at position 627 in PB2, instead K702R was present which is linked to increased activity of the viral polymerase in mammalian hosts and found less frequently (~7%) among bird isolates (EFSA AHAW Panel and ECDC, 2025).

#### 2.3.4 Human A(H10N3) cases

Between 29 November 2025 and 27 February 2026, one human case was reported in the Guangdong Province in China (CHP, online-d; WHO, online-f). The case was a 34-year-old man, who developed symptoms on 29 December 2025. The patient was hospitalised and is, at the time of writing, in a stable condition. Exposure to live poultry prior to symptom onset was reported. No sequences were available for this A(H10N3) human case.

Since the first report in 2021 and as of 27 February 2026, a total of seven cases have been reported globally, all in China (different provinces) and with no associated deaths. All reported cases were in adults. All but one of the cases had a severe or critical infection and reported exposure to live animals or a contaminated environment.

#### 2.3.5 Additional information relevant for public health and international risk assessments

Following outbreaks in fur farms in 2023, Finland was the first country in the EU/EEA to make H5 vaccination available for individuals occupationally at risk. The results of their study to evaluate the immune responses induced by the vaccines in occupationally exposed individuals have recently been published (Liedes et al., 2026). They showed that the vaccine raised antibodies capable of neutralising two heterologous clade 2.3.4.4b A(H5N1) strains (from outbreaks at fur farms in Finland and in dairy cattle in the US) and cellular immunity.

During the reporting period (29 November 2025–27 February 2026), the results of several studies conducted in North America evaluating H5N1 cross-reactive immunity were published (Li et al., 2025; Bonifaz et al., 2026; Daniel et al., 2026; Singh et al., 2026; Skowronski et al., 2026). They showed that antibodies raised after natural infection with seasonal influenza virus or vaccination with adjuvanted vaccines might provide some level of cross-reactivity. In addition, several detailed reports of clade 2.3.4.4b A(H5N1) human cases detected in Canada and the USA were published (Hatta et al., 2025; Higerd-Rusli et al., 2025; MacBain et al., 2026).

In vitro studies remain important for the assessment of clade 2.3.4.4b A(H5N1) viruses to understand their capacity to infect mammals and to evaluate the zoonotic potential. The results of two studies looking at the in vitro capacities of the virus to attach and infect human respiratory epithelium were recently published. The first study compared several clade 2.3.4.4b A(H5N1) viruses with a 2005 clade 2.1.3.2 A(H5N1) virus and showed that better attachment and infection by 2.3.4.4b viruses might be associated with a broader antiviral response, and not with an increased polymerase activity (Bauer et al., 2026). The second study compared two genotypes of clade 2.3.4.4b A(H5N1) viruses responsible for human cases in the USA and showed that the D1.1 genotype could replicate to higher titres than the B3.13 genotype in the human respiratory tract, without detecting differences in the cytokine profiles following cell infection (Zhang et al., 2025).

In the United Kingdom, a survey was conducted to assess the perceived risk of avian influenza among people having regular contact with birds (Whitlow et al., 2026). The risk of avian influenza infection on their own health was perceived as low by the respondents, while the risk on bird health was rated as high by individuals having contacts with birds in large flocks. The biosecurity measures most likely to be used were hand washing and footwear disinfection.

Sequence analysis of A(H5N1) viruses circulating in Nepal in 2023 highlighted the presence of both clades 2.3.4.4b and 2.3.2.1a in poultry (Koirala et al., 2025). The 2.3.2.1a virus was found to be a reassortant containing the NS, PB1, PB2 and PA segments from a 2.3.4.4b virus. The reassortant virus was found to have a high genetic identity with viruses of clade 2.3.2.1a that were responsible for cases in humans and cats in India in 2024–2025.

The Food and Agriculture Organization of the United Nations (FAO), WHO and WOAH tripartite assessment of the public health risk posed by influenza A(H5N1) viruses remains unchanged and there has been no update of the assessment published on 28 July 2025. The risk of infection is considered low for the general public and low-to-moderate for occupationally exposed individuals (FAO, WHO and WOAH, online). In the latest summary and risk assessment of 'Influenza at the human-animal interface' (WHO, online-g), WHO's assessment of the risk to public health for the currently known avian influenza viruses has not changed and is still considered to be low.

During the reporting period, the US Centers for Disease Control and Prevention (CDC) assessment of the public health risk posed by avian influenza A(H5) viruses has not changed and is currently considered low in the USA (CDC, online).

Similarly, during the reporting period, the United Kingdom Health Security Agency (UK HSA) risk assessment of HPAI A(H5) to the general public, as available in 'Bird flu (avian influenza): latest situation in England' updated on 22 February 2026, has remained unchanged and is still considered to be very low (GovUK, online-d).

#### 2.3.6 ECDC risk assessment

ECDC's assessment of the risk of human infection with HPAI A(H5N1) clade 2.3.4.4b viruses remains unchanged. Overall, ECDC assesses the risk posed by HPAI A(H5N1) clade 2.3.4.4b viruses currently circulating in animals in Europe to be low for the general public in the EU/EEA, and low-to-moderate for those occupationally or otherwise exposed to infected animals or contaminated environments. The assessment considers the risk of infection at population level in the EU/EEA, taking into account the likelihood of human infection and transmission and the potential impact of such an infection, based on ECDC's framework for risk assessments (ECDC, 2019).

The risk assessment was informed by available evidence related to the transmission, prevalence, and characteristics of A(H5N1) viruses currently circulating in animals in Europe, as well as knowledge acquired from human cases of influenza A(H5N1) reported globally. This evidence is set out below.

The likelihood of human infection with HPAI A(H5N1) clade 2.3.4.4b viruses in the EU/EEA:

- Despite the extensive circulation of avian influenza A(H5N1) virus in animal populations in Europe this autumn and in the past few years, with frequent opportunities for human exposure, there have been no confirmed cases of A(H5N1) clade 2.3.4.4b infection in humans in the EU/EEA, and transmission from infected animals to humans remains a rare event globally.
- In Europe, mutations associated with viral adaptation to mammalian hosts have sporadically been identified in sequences of A(H5N1) clade 2.3.4.4b viruses from mammals and birds. However, circulating viruses retain a binding preference for α2–3 sialic acid (avian-type) receptors and are still considered to be avian-like. There is currently no evidence of the viruses being more capable of infecting humans or having the ability to transmit between humans. Further understanding of the implications of such mutations for mammalian adaptation, infection and transmission will help in assessing any change in the associated risk to humans.
- Sporadic cases of infection with avian influenza A(H5N1) virus have been reported in humans globally, often with a history of unprotected exposure to poultry, cattle or contaminated environments. The only A(H5N1) human case reported globally between 29 November 2025 and 27 February 2026 had documented exposure to poultry.
- To date, there has been no evidence of human-to-human transmission of influenza A(H5N1) clade 2.3.4.4b viruses.

The impact of human infection with HPAI A(H5N1) clade 2.3.4.4b viruses:

- Historically, clinical presentations of individuals infected with A(H5N1), including clades other than 2.3.4.4b, have ranged from asymptomatic or mild (such as conjunctivitis and upper respiratory tract symptoms) to severe illness resulting in death, with a case fatality estimated at 48% since 2003. However, this figure is only based on reported cases and may be overestimated.
- The majority of human cases of infection with influenza A(H5N1) clade 2.3.4.4b reported in the USA since March 2024, and the human case reported in the United Kingdom in January 2025, experienced mild symptoms, such as conjunctivitis or mild respiratory illness. Between 29 November 2025 and 27 February 2026, no new cases of human infection with an A(H5N1) clade 2.3.4.4b virus were reported globally.
- The reasons for the variation in outcome following infection with influenza A(H5N1) virus are likely to be multi-factorial and may be attributed to the virus genotype, duration of exposure, viral load, transmission route, individual health status, personal protective measures taken, and medical treatment provided (FAO, WHO and WOAH, online). The detection of several mild cases of A(H5N1) virus infection and seropositive individuals without a history of clinical symptoms in the USA suggests that mild and asymptomatic cases may be more common than previously reported for A(H5N1). Targeted surveillance of individuals exposed to infected animals is therefore recommended (EFSA AHAW Panel and ECDC, 2025).
- Most of the circulating A(H5N1) clade 2.3.4.4b viruses detected in Europe remain susceptible to antiviral medicines available to treat humans, including adamantanes, neuraminidase inhibitors (e.g. oseltamivir) and polymerase acidic inhibitors (e.g. baloxavir marboxil).

Sporadic human infections with avian influenza viruses are likely to continue occurring in areas where the virus is prevalent and individuals have unprotected contact with infected animals or their contaminated environments. Circumstances or conditions that increase mammalian or human exposure to avian influenza viruses may lead to spillover events and further transmission, increasing the probability of avian influenza viruses adapting to mammals (EFSA AHAW Panel and ECDC, 2025). Given the ongoing extensive transmission of A(H5N1) in wild birds and poultry, and in some mammals in certain settings, viral evolution through mutations or reassortment may occur, which could change the current assessment. Therefore, ECDC is regularly reviewing the risk assessment, taking into consideration any new developments or information that becomes available.

## 3 Conclusions

### 3.1 Birds

- There was a higher rate of HPAI virus detections in waterfowl during the current reporting period, and during the 2025–2026 epidemiological year overall, compared to previous years. Possible reasons for this high rate include more efficient transmission of the novel genotype EA-2024.DI.2.1, decreased flock immunity, and/or a higher proportion of juvenile birds in the waterfowl population. During the last weeks of the current reporting period, there was a declining trend in detections, which coincides with the end of the autumn migration of waterfowl species.
- Mute swans, in which mortality was reported from several European countries, and different wild goose species continued to be affected in high numbers. There was also a substantial number of Eurasian buzzards involved in the current reporting period.
- HPAI virus detections in common cranes stopped, after a marked peak, coinciding with the end of their autumn migration.
- The geographical distribution of HPAI virus detections in wild birds during the current reporting period corresponds to important wintering areas in central-western Europe and largely overlaps with those in domestic birds.
- In the coming months, HPAI virus detections in wild waterfowl are expected to decrease, as migratory species are continuously leaving their wintering areas.

- Mostly primary outbreaks following indirect contact with wild birds were reported in domestic birds in Europe during the current reporting period. Despite the high number and widespread occurrence of HPAI virus detections in wild birds in Europe, and the associated high risk posed to poultry, some affected establishments still provided outdoor access.
- During the current reporting period HPAI viruses were detected in 14 establishments keeping vaccinated ducks in France, where expression of clinical signs and mortality varied. This underscores the importance of implementing multiple components of post-vaccination surveillance, including passive and active surveillance components. These HPAI outbreaks reported in vaccinated ducks in France show that vaccination alone is not enough to prevent HPAI outbreaks in poultry establishments located in areas with extensive virus circulation in the local wild bird population, but that it needs to be combined with strict and systematic biosecurity measures. The fact that no or only limited secondary spread between establishments was observed differs from previous epidemiological years when vaccination had not been practised (i.e 2020–2021 or 2021–2022) and possibly suggests that the vaccination strategy in place, in addition to surveillance and biosecurity measures, has been effective in reducing the risk of spread from the affected establishments.
- As in previous years, multiple zoos and similar establishments exhibiting captive birds to the public were affected by HPAI viruses.
- No new HPAI A(H5N5) virus detections were reported in Europe or elsewhere via ADIS and WOAH-WAHIS during the current reporting period. However, there was a single detection of HPAI A(H5N2) virus in a barnacle goose in Sweden.
- Most clade 2.3.4.4b HPAI A(H5N1) viruses identified in Europe since October 2025 belong to the genotype EA-2024.DI.2.1, which was also responsible for the mass mortality events reported in common cranes in several European countries. This genotype has been identified all over Europe, including Iceland, located along the East Atlantic Flyway. As this flyway also extends to Greenland and the Canadian Arctic, Iceland may represent an important connecting site for the possible virus movement from northern Europe to North America. Outside Europe, EA-2024.DI.2.1 was identified in Kazakhstan.
- Since October 2025, markers of mammalian adaptation in the PB2 protein have been detected in less than 0.5% of European viruses collected from birds.
- In general, and as previously reported, low numbers of wild birds infected with HPAI viruses were reported from outside Europe via official notifications to WOAH. Despite these low numbers, HPAI viruses were increasingly reported in snow and Canada geese from North America. In India, mortality in crows was reported in several states.

### 3.2 Mammals

- During the current reporting period, there was a slight increase in the number of HPAI A(H5N1) virus detections in mammals in Europe, predominantly in wild carnivores, while no new HPAI A(H5N5) virus detections were reported in Europe or elsewhere.
- The number of HPAI virus detections in sick and dead mammals can still be considered low given the high circulation of HPAI virus in the wild bird population and the resulting high contamination of the environment in the current epidemiological year.
- In the Netherlands, the first potential spillover of HPAI A(H5N1) virus from wild birds into dairy cattle in Europe was suggested by serology. The event demonstrates that similar spillover events as observed in the USA may also occur in Europe with a locally circulating HPAI A(H5N1) virus strain.

- In the USA, the rate of new HPAI virus detections in dairy cattle further declined, but three additional spillover events from wild birds, involving a different genotype (D1.1), were documented in three different states since the beginning of 2026. These events confirm that spillover of HPAI virus into dairy cattle can happen repeatedly, and with different genotypes.
- Two new mammal species, the island fox and the northern elephant seal, tested positive for HPAI virus for the first time in the USA.
- Genetic data available for 20 A(H5N1) viruses collected from wild and domestic mammals in Europe indicate that they belong to clade 2.3.4.4b, genotype EA-2024-DI.2.1. Of those, 90% are distinguishable from genetically related viruses identified in avian species by the presence of molecular markers of adaptation to mammals in the PB2 protein.

#### 3.3 Humans

- Despite the widespread occurrence of HPAI A(H5N1) in wild birds, poultry and some mammals in recent years, with many potential exposures of humans to infected animals, there have been no confirmed human cases of influenza A(H5N1) in the EU/EEA.
- The majority of human infections with avian influenza viruses reported since 1997 have been associated with unprotected exposure to poultry, live poultry markets, or contaminated environments. In addition, since March 2024, there have been 41 human cases of influenza A(H5) reported following exposure to dairy cattle infected with A(H5N1) virus. To date, there has been no evidence of sustained human-to-human transmission.
- In the reporting period from 29 November 2025 to 27 February 2026, there was only one human case of avian influenza A(H5N1) infection reported globally. The case had documented exposure to poultry.
- With the extensive circulation of avian influenza viruses in bird populations globally, sporadic transmission to humans is likely to continue in settings where people have unprotected exposure to infected animals or their environment.

### 4 Options for response

#### 4.1 Birds

- Biosecurity measures in domestic bird establishments and compliance thereof should be optimised and maintained at high level both during the production cycle and culling operations. This is particularly the case for establishments under the same ownership or located in high-density poultry areas.
- Housing orders for domestic birds are strongly recommended in high-risk areas, such as those where HPAI virus is circulating in wild birds or mass mortality events in wild birds have been described.
- While vaccination of poultry can substantially reduce the risk of outbreaks taking place in vaccinated compared to unvaccinated flocks, it does not provide complete protection on its own. Therefore, as it would be the case for unvaccinated flocks, vaccination should be accompanied by strict biosecurity measures and adequate surveillance.
- Surveillance in domestic birds should be enhanced to ensure early detection of infected poultry establishments. This is particularly the case for areas or production systems where secondary spread has been observed.
- During high-risk periods in high-density poultry areas, a specific control strategy may be implemented to reduce the number of establishments stocked with species that are highly susceptible to HPAI (e.g. turkeys), thereby reducing the secondary spread of the disease.

- Clearing up wild bird carcasses should be considered during periods of extensive wild bird mortality due to HPAI to reduce the level of environmental virus contamination and the risk of further spread among wild and domestic animals. This includes wild bird mortality occurring in areas near poultry establishments or where animals are grazing.
- Surveillance activities in wild birds should be enhanced, particularly in wetland areas and at migratory stopover sites in and outside Europe. Given the low level of HPAI-associated mortality in wild waterfowl, particularly wild ducks, relative to the high level of virus circulation in apparently healthy individuals, there is a greater need to sample apparently healthy waterfowl ('active surveillance') in addition to waterfowl found dead ('passive surveillance') in order for avian influenza surveillance in wild birds to act as a more sensitive early warning system for the presence of HPAI in a geographical area. This 'active surveillance' is particularly relevant in autumn at key sites along the flyways of migratory waterfowl.
- Zoos and other establishments exhibiting captive birds to the public, as well as wildlife rescue/rehabilitation centers and bird shelters, should be part of surveillance activities and implement adequate biosecurity to protect both animal and public health.
- EFSA's Bird Flu Radar may be consulted to monitor the probability of HPAI virus introduction in wild bird populations over space and time: https://app.bto.org/hpai
- For wild birds, options for response include accurate and comprehensive recording of HPAI-associated mortality events (to estimate the impact on wild bird populations) and preventing disturbance of areas undergoing HPAI outbreaks to reduce virus spread (e.g. hunting activities, tourism, leisure activities, use of drones).
- Artificial feeding of wild birds should be avoided during high-risk periods to reduce the level of crowding of these species.
- It remains important to continuously monitor LPAI viruses of the A(H5) and A(H7) subtypes in wild and domestic birds, and introductions of these subtypes into poultry establishments, as these subtypes can mutate into their highly pathogenic forms once circulating in poultry.
- Strengthening the genetic characterisation of viruses is recommended to quickly identify molecular markers of adaptation to mammals, which could indicate potential virus circulation in mammals and subsequent mammal-to-avian transmission. Viruses carrying such mutations may have an increased zoonotic potential and their biological characteristics should be further evaluated to assess the actual impact of the acquired mutations. To serve as an effective early warning system, sequences should be generated and shared in public databases as promptly as possible. Genetic data are also instrumental to track the virus spread, support epidemiological investigations in the distinction between primary and secondary outbreaks, and identify novel incursions of viruses that may represent a threat for human or animal health.

### 4.2 Mammals

- Increased virological and serological surveillance of HPAI viruses in wild (e.g. red foxes) and free-roaming domestic carnivores (e.g. cats and dogs) in areas with high HPAI virus circulation continues to be recommended to monitor both the level of virus infection in these species and the risk of emergence and transmission of mammalian-adapted viruses. Surveillance should also focus on domestic and farmed mammals exposed to highly contaminated environments (e.g. fur animals, ruminants, pigs and camelids), in close contact with HPAI-affected poultry or wildlife, or present in mixed-species establishments (e.g. backyard farms, petting farms, zoos). Research to investigate the role of mammals in maintaining HPAI viruses and driving their evolutionary dynamics is recommended.

- In light of the first potential spillover of HPAI A(H5N1) virus from wild birds into dairy cattle in Europe, testing of/surveillance in ruminants is recommended when a combination of factors is observed, such as the manifestation of overt and unresolved clinical signs typically associated with HPAI virus infection in ruminants (e.g. undiagnosed severe decrease in milk production and presence of darker, thickened milk), but also the occurrence of HPAI virus infection in other domestic, peridomestic or wild animals in/around farms keeping ruminants. In addition, HPAI should be considered as a differential diagnosis in cases of undiagnosed or unresolved compatible clinical signs during periods of HPAI virus circulation in the area where ruminants are kept.
- In pigs, swine influenza viruses are widely circulating (Mena-Vasquez et al., 2025). Moreover, multiple spillover events of seasonal influenza viruses from humans to swine and vice versa have been frequently demonstrated. Pigs may play an important role in the emergence of new reassortant viruses with unknown biological properties and possible increased pandemic potential. The role of pigs as mixing vessel is well known; therefore, surveillance of avian influenza viruses in pigs in close contact with HPAI-affected poultry or wildlife is recommended.
- In order to be prepared for a possible rapid increase in the number of tests to be carried out, national reference laboratories should consider purchasing tests and reagents to perform virological and serological diagnostic activities on mammals, including ruminants. Maintaining contact with the European Union Reference Laboratory (EURL) is recommended to ensure the use of appropriate virological and serological tests. Virological and serological investigations in mammals should increase knowledge on HPAI and LPAI viruses that pose a potential zoonotic risk and assess the extent to which these viruses circulate in these species in order to guide future surveillance activities.
    - In some cases, the diagnostic performance of commercial ELISA kits has changed due to updates in the protocols recommended by the manufacturers. Comparison and assessment of the performance of protocols for different mammalian species is in progress. Therefore, it is advisable to rely on the EURL indications for serological surveillance of HPAI virus infections in mammals (contact details are available on the EURL website: https://www.izsvenezie.com/reference-laboratories/avian-influenza-newcastle-disease/)
- Pets and other captive mammals should not be fed with raw meat, raw pet food, or other raw animal products (e.g. raw milk) from sources that have not been adequately controlled for possible HPAI virus contamination. The risk associated with feeding contaminated raw pet food (based on poultry) to domestic cats, a practice that has been reported in several countries in the world, including in the EU, stresses the importance of highly sensitive surveillance systems for early detection, and of the removal and destruction of infected poultry flocks as well as of contaminated animal products.
- At times of high HPAI virus circulation in an area, it is recommended to keep pets indoors or on a leash.
- In high-risk areas during high-risk periods, private veterinarians should be adequately informed and encouraged to participate in the surveillance of potential spillover events into pet carnivores such as domestic cats or dogs, and ferrets. If suspect clinical signs are observed (e.g. neurological or respiratory signs), these animals should be tested for avian influenza.

- More accurate and timely reporting of HPAI virus detections in mammals is recommended in a way that reliable numbers of infected animals could be used as quantitative information for risk assessment.

### 4.3 Humans

- The risk of human exposure to avian influenza viruses can be reduced by implementing some of the prevention measures suggested below.
    - Use of appropriate personal protective equipment when in contact with potentially infected animals or highly contaminated environments.
    - Implementation of adequate biosecurity and biosafety measures at occupational sites where there is increased risk of exposure in order to reduce the risk of zoonotic infection and ensure safe handling of potentially contaminated biological materials.
    - Provision of information and guidance to raise awareness among people at risk of exposure and indicate how the risk can be mitigated. Guidance should be tailored to specific occupational groups, or individuals engaged in recreational activities where additional measures may be beneficial. Recommendations for personal protective measures should consider the working environment, tasks performed, routes of exposure and environmental factors.
    - Recommendations to the general public and those keeping backyard or commercial poultry are to avoid contact with sick or dead birds and wild animals. If they find dead animals, they should inform the relevant authorities in order to ensure safe removal and further investigation.
    - Detections of HPAI in apparently healthy wild ducks (EFSA and EURL, 2025) highlight the importance of precautionary measures when handling wild birds, even in the absence of clinical signs. Information to increase awareness of the risk of infection from animals and set out the appropriate preventive measures should also be made available to those in contact with wild birds, such as hunters and wildlife workers.
- People exposed to animals with suspected or confirmed avian influenza virus infection while not wearing appropriate personal protective equipment should be monitored for symptoms for 10–14 days after the last exposure and tested if symptoms develop. The situation should be assessed on a case-by-case basis, depending on the level of exposure. Further information on testing, follow-up and management of individuals with exposure and confirmed infection can be found in ECDC's guidance 'Testing and detection of zoonotic influenza virus infections in humans' (ECDC, 2022); 'Investigation protocol for human exposures and cases of avian influenza' (ECDC, 2023) and the ECDC/EFSA guidance 'Coordinated One Health investigation and management of outbreaks in humans and animals caused by zoonotic avian influenza viruses' (ECDC and EFSA, 2025).
- Countries should remain vigilant for potential human cases of avian influenza, especially in geographical areas where the virus is known to circulate in poultry, wild birds, or other animals.
- In areas with ongoing avian influenza outbreaks in animals, healthcare workers (including primary care providers) should be made aware of the epidemiological situation in animal populations and the range of symptoms associated with avian influenza infection in humans. They should ask patients with symptoms compatible with zoonotic avian influenza infection (e.g. symptoms of upper or lower respiratory tract infection, but also non-respiratory symptoms, such as conjunctivitis, gastrointestinal, or neurological symptoms) about their history of exposure to animals (ECDC, 2024).

- Detections of human cases of zoonotic avian influenza through surveillance systems for seasonal influenza highlight the importance of typing and subtyping samples. All influenza-positive specimens collected through seasonal influenza sentinel surveillance from both primary and secondary care sources should be typed and subtyped.
- During periods of high seasonal influenza virus circulation (typically during the winter in EU/EEA countries), exhaustive subtyping of influenza type A-positive samples might not be possible. Testing and subtyping specifically for avian influenza virus could be done on a risk-based approach, depending on the epidemiological situation in animal populations and focusing on individuals with known exposure to potentially infected animals, or cases with severe respiratory signs or neurological symptoms of unknown aetiology. Further guidance is available in ECDC's technical report on targeted surveillance to identify human infections with avian influenza virus during the influenza season (ECDC, 2024).
- Genetic changes in avian influenza viruses that may alter their zoonotic potential (increase capacity to infect humans or increase transmissibility) or their susceptibility to antivirals available to treat humans should be monitored (EFSA AHAW Panel and ECDC, 2025). Avian influenza viruses detected in humans should be sequenced and the sequence shared in public databases in a timely manner.
- Vaccination against seasonal influenza should be offered to individuals who are occupationally exposed to avian influenza viruses to limit infection with seasonal influenza in humans and reduce the risk of co-infection with human and avian subtypes of influenza and the possibility of genetic reassortment between viruses (EFSA AHAW Panel and ECDC, 2025).
- Zoonotic avian influenza A(H5) vaccination in individuals occupationally or otherwise routinely exposed to infected animals or contaminated environments could be considered as a complementary preventive measure. While data on immune response induction are available, there is limited information on reduction in infection or onward transmission and protection against severe clinical disease (EFSA AHAW Panel and ECDC, 2025). Consequently, at present there is insufficient evidence to support a broad recommendation for their use across all EU/EEA countries (ECDC, 2025).
- The options for response are based on current available evidence, the epidemiological situation and the risk assessment for the EU/EEA. Recommended measures may need to be adapted if the epidemiological situation changes, or new evidence becomes available.

## Appendix A – Terms of Reference of the joint EFSA-ECDC mandate (M-2024-00009) accepted in March 2024

### A.1. Background and Terms of Reference as provided by the requestor

Avian influenza is an infectious viral disease in birds, including domestic poultry. Infections with avian influenza viruses in poultry cause two main forms of that disease that are distinguished by their virulence. The low pathogenic (LPAI) form generally only causes mild symptoms, while the highly pathogenic (HPAI) form results in very high mortality rates in most poultry species. That disease may have a severe impact on the profitability of poultry farming.

Avian influenza is mainly found in birds, but under certain circumstances infections can also occur in humans even though the risk is generally very low.

More than a decade ago, it was discovered that virus acquired the capability to be carried by wild birds over long distances. This occurred for the HPAI of the subtype A(H5N1) from South-East and Far East Asia to other parts of Asia, Europe and Africa as well as to North America. In the current epidemic the extent of the wild bird involvement in the epidemiology of the disease is exceptional.

The evolution of the HPAI epidemiological situation with high number of birds and new mammalian species affected is prompting response by both animal health and public health authorities in EU Member States and indicates the need for enhanced preparedness and prevention. Given the mammalian adaptation mutations detected in certain circulating viruses, the infection of mammals in fur farms, as well as an outbreak amongst cats extending the animal/human interface along with the suspicion of events of mammal-to-mammal transmission, animal health and public health authorities are currently working on addressing these challenges. In that context, they are developing or adapting their tools for epidemiological investigations, strengthening their collaboration issuing new emergency national legislations as well as adapting their surveillance guidance and programmes in the light of the upcoming seasonal flu season. The situation and actions above have been taken in reaction to:

- ECDC's current risk assessment that focuses on the immediate risk of avian influenza for human health fulfilling ECDC's new mandate and the Regulation (EU) 2022/2371[7] aiming to prevent and prepare for cross-border health threats, including epidemics; and
- EFSA's latest scientific opinions and reports coupled by measures taken (i.e., surveillance, prevention and control measures) under the Animal Health Law (i.e., Commission Delegated Regulation (EU) 2020/689[8] and Commission Delegated Regulation (EU) 2020/687[9]).

[7] Regulation (EU) 2022/2371 of the European Parliament and of the Council of 23 November 2022 on serious cross-border threats to health and repealing Decision No 1082/2013/EU. OJ L 314, 6.12.2022, p. 26–63.
[8] Commission Delegated Regulation (EU) 2020/689 of 17 December 2019 supplementing Regulation (EU) 2016/429 of the European Parliament and of the Council as regards rules for surveillance, eradication programmes, and disease-free status for certain listed and emerging diseases. OJ L 174, 3.6.2020, p. 211–340.
[9] Commission Delegated Regulation (EU) 2020/689 of 17 December 2019 supplementing Regulation (EU) 2016/429 of the European Parliament and of the Council as regards rules for surveillance, eradication programmes, and disease-free status for certain listed and emerging diseases. OJ L 174, 3.6.2020, p. 211–340.

In view of the One Health nature of this subject, and pursuant to Article 3 of Regulation (EU) 2022/2370[10] and Articles 29 and 31 of Regulation (EC) No 178/2002[11], the European Centre for Disease Prevention and Control (ECDC) and the European Food Safety Authority (EFSA) are requested to carry out the following task:

To provide regular quarterly scientific reports, updating on the avian influenza situation within the Union and worldwide, to the Commission by:

1 Analysing the epidemiological data on HPAI and LPAI from Member States and describe the evolution of virus spread from certain regions towards the EU and in case of significant changes in the epidemiology of avian influenza;
2 Analysing the temporal and spatial pattern of HPAI and LPAI, as appropriate, in poultry, captive and wild birds, kept and wild mammals, as well the risk factors involved in the occurrence, spread and persistence in the EU of the avian influenza virus in and at the interface of these animal populations with specific attention to zoonotic risks;
3 Describing the options for adapting preparedness, prevention, and control measures, based on the finding from point 1) and 2).

### A.2. Interpretation of the Terms of Reference

In reply to the TORs above, this Scientific Report gives an overview of the HPAI virus detections in poultry, captive and wild birds, as well as in mammals, in Europe and worldwide between 6 September and 28 November 2025, as reported by Member States and third countries via ADIS or WOAH-WAHIS. In addition, LPAI virus detections of specific relevance are included, and possible actions for preparedness in the EU are discussed based on the situation worldwide. Member States and other European countries where HPAI outbreaks have occurred in poultry submitted additional epidemiological data to EFSA, which have been used to analyse the characteristics of the affected poultry establishments.

However, it was not possible to collect data for a comprehensive risk factor analysis on the occurrence and persistence of HPAI viruses within the EU. Risk factor analysis requires not only case-related information but also data on the susceptible population (e.g. location of establishments and population structure), which should be collected in a harmonised manner across the EU. Limitations in data collection, reporting and analysis were explained in the first avian influenza overview report (EFSA, ECDC and EURL, 2017).

This report mainly describes information that has become available since the publication of the EFSA report for the period June to September 2025 (EFSA, ECDC and EURL, 2025b) and that might affect the interpretation of risks related to avian influenza introduction and/or spread in Europe.

[10] Regulation (EU) 2022/2370 of the European Parliament and of the Council of 23 November 2022 amending Regulation (EC) No 851/2004 establishing a European centre for disease prevention and control. OJ L 314, 6.12.2022, p. 1–25.
[11] Regulation (EC) No 178/2002 of the European Parliament and of the Council of 28 January 2002 laying down the general principles and requirements of food law, establishing the European Food Safety Authority and laying down procedures in matters of food safety. OJ L 31, 1.2.2002, p. 1–24.

## Appendix B – Data and Methodologies

### B.1. Data on animals

#### B.1.1. Overview of avian influenza outbreaks in Europe

For this report, data on HPAI outbreaks reported in Europe between 29 November 2025 and 27 February 2026 and submitted by Member States and other European countries via ADIS, were taken into account. Data extraction was carried on 27 February 2026. WOAH-WAHIS was consulted to complement the information for European countries not notifying HPAI via ADIS (United Kingdom (excluding Northern Ireland)). Additionally, countries affected by HPAI were asked to provide EFSA with more detailed epidemiological information on HPAI outbreaks in poultry. This information included details on the poultry species and production systems affected by HPAI, which were supplied in form of additional variables to complement the data reported via ADIS. All data collected are provided in Annex B. In addition, the information European countries affected by avian influenza presented to the Standing Committee on Plants, Animals, Food and Feed (SCOPAFF), and the evidence on avian influenza outbreaks provided in the info notes from affected countries to the EC, were consulted to extract relevant information reported in Section 2.1.1. The presentations delivered at the SCOPAFF meetings are available on the EC website (European Commission, online).

Wild bird species have been categorised according to Table A.2, and the common and scientific names of wild bird species described in this report for Europe are reported in Table A.3 (both in Annex A). For mammals, the common and scientific names of the species involved are listed in Table 3. The GISAID's EpiFlu™ database was accessed to download newly released avian influenza virus sequences.

The annexes to this Scientific Report are available here: https://doi.org/10.5281/zenodo.18978040

#### B.1.2. Overview of avian influenza outbreaks in other countries not reporting via ADIS

Data from WOAH-WAHIS on HPAI A(H5) and A(H7) in domestic and wild birds were used to describe and map the geographic distribution of HPAI virus detections in domestic and wild birds in other regions of the world based on their observation dates. Data were retrieved from WOAH-WAHIS and extracted on 27 February 2026. They were used and reproduced with permission. WOAH bears no responsibility for the integrity or accuracy of the data contained therein, not limited to: any deletion, manipulation, or reformatting of data that may have occurred beyond its control.

#### B.1.3. Genetic characterisation of avian influenza viruses: description of the nomenclature of the HPAI A(H5) viruses used in the document

The HA gene of clade 2.3.4.4 A(H5) viruses has rapidly evolved since the most recent official update of the nomenclature of the A/goose/Guangdong/1/1996-lineage H5Nx virus (Smith et al., 2015). This clade emerged in China in 2008 and since then has acquired various neuraminidase subtypes, including N1, N2, N3, N4, N5, N6 and N8, by reassortments with other avian influenza viruses from different regions, and has evolved into several subgroups. While a revised nomenclature of clade 2.3.4.4 viruses is pending, in previous reports the genetic clustering described in 2018 by Lee and co-authors, who recognised four groups (a–d) within clade 2.3.4.4 (Lee et al., 2018), was used. Recently, an update to the unified nomenclature for clade 2.3.4.4 A(H5) viruses has been proposed by the WHO, and eight genetic groups (a–h) have been recognised. To align the nomenclature system between international organisations, this classification has been adopted for this report.

Based on this proposed clustering, A(H5) viruses of clades 2.3.4.4a and d–h have mainly been circulating in poultry in Asia, while clades 2.3.4.4b and 2.3.4.4c have spread globally through wild bird migrations during 2014–2015 (2.3.4.4c) and from 2016 to the present day (2.3.4.4b). A list with the distribution of the different genetic clades reported by countries globally from birds, humans and the environment has been published by WHO in February 2023 (WHO, 2023).

### B.2. Data on humans

Data on the number of human cases caused by infection with avian influenza viruses were collected by ECDC. As part of epidemic intelligence activities at ECDC, multiple sources are scanned regularly to collect information on laboratory-confirmed human cases. Data were extracted and line lists developed to collect case-based information on virus type, date of disease onset, country of reporting, country of exposure, sex, age, exposure, clinical information (hospitalisation, severity) and outcome. All cases included in the line list and mentioned in the document have been laboratory-confirmed. Data are continuously checked for double entries and validity. The data on human cases cover the full period since the first human case was reported. Therefore, data on human cases refer to different time periods. Relevant information on human infections, risk factors, and the results from studies on infection and transmission with relevance for human health are included.

#### B.2.1. Method for phylogenetic and mutation analysis

The GISAID's EpiFlu™ Database (Shu and McCauley, 2017) was accessed on 27 February 2026 to retrieve sequences from human cases of avian influenza reported since 29 November 2025. In cases where the same strain had multiple submissions, the ones with the most complete segment data were chosen. Sequences placed under an embargo were excluded from any analysis. Mutations were retrieved using FluMut (Github izsvenezie-virology, online-a, b) with the FluMutDB v6.4 mutation database (https://github.com/izsvenezie-virology/FluMutDB). Mutations found in less than 10% of the background sequences were considered significant. Clade information was retrieved from GISAID or assigned according to specified literature. Genin2 (https://github.com/izsvenezie-virology/genin2) and GenoFLU (Youk et al., 2023) were used to assign genotypes.

## Annex A – Data on HPAI virus detections in wild birds

Annex A includes additional figures and tables on HPAI virus detections in wild birds. The Annex is available on the EFSA Knowledge Junction community on Zenodo at: https://doi.org/10.5281/zenodo.18978040

## Annex B – Characteristics of the HPAI-affected poultry establishments

Annex B includes the HPAI outbreaks in poultry for which additional data on the characteristics of the affected poultry establishments were collected and provided by reporting countries during the current reporting period. It also includes data on outbreaks that occurred too close to the publication of the previous report and are therefore included here. The Annex is available on the EFSA Knowledge Junction community on Zenodo at: https://doi.org/10.5281/zenodo.18978040

## Annex C – Control measures applied in Hungary to reduce secondary spread

Annex C details the control measures applied in Hungary to reduce secondary spread between poultry establishments. The Annex is available on the EFSA Knowledge Junction community on Zenodo at: https://doi.org/10.5281/zenodo.18978040

## Annex D – Acknowledgements

All genome sequences and associated metadata linked in the below dataset are published in GISAID's EpiFlu™ database (Shu and McCauley, 2017). To view the contributors of each individual sequence with details such as accession number, virus name, collection date, originating lab and submitting lab, and the list of authors, visit: https://doi.org/10.55876/gis8.260310sv and https://doi.org/10.55876/gis8.260310py.

**The EFSA Journal is a publication of the European Food Safety Authority, an agency of the European Union.**
